# Prognostic significance of nutritional status for neurological and functional recovery after cervical spinal cord injury

**DOI:** 10.1371/journal.pone.0353302

**Published:** 2026-07-07

**Authors:** Momo Irie, Kazuya Yokota, Tomoya Matsunaga, Osamu Kawano, Muneaki Masuda, Kensuke Kubota, Yuto Ariji, Ryuichiro Koga, Hiroaki Sakai, Takeshi Maeda, Yasuharu Nakashima, Tetsuo Hayashi

**Affiliations:** 1 Department of Orthopaedic Surgery, Japan Organization of Occupational Health and Safety, Spinal Injuries Center, Iizuka, Fukuoka, Japan; 2 Department of Orthopaedic Surgery, Graduate School of Medical Sciences, Kyushu University, Higashiku, Fukuoka, Japan; 3 Department of Rehabilitation Medicine, Japan Organization of Occupational Health and Safety, Spinal Injuries Center, Iizuka, Fukuoka, Japan; 4 Department of Orthopedic Surgery, Japan Organization of Occupational Health and Safety, Hokkaido Spinal Cord Injury Center, Bibai, Hokkaido, Japan; 5 Department of Rehabilitation Medicine, Fukushima Medical University School of Medicine, Hikarigaoka, Fukushima, Japan; Shuguang Hospital, CHINA

## Abstract

Cervical spinal cord injury (SCI) often results in severe motor and sensory deficits, leading to substantial impairment in activities of daily living. Malnutrition is common after SCI and may adversely affect recovery; however, the association between early nutritional status and subsequent neurological and functional recovery remains unclear. This study examined the association between early nutritional status and neurological and functional recovery after acute traumatic cervical SCI. Ninety-one patients admitted within 72 hours of injury and followed for at least 6 months were retrospectively analyzed. Neurological function was assessed using the International Standards for Neurological Classification of Spinal Cord Injury (ISNCSCI) total motor score, and functional independence was evaluated using the Spinal Cord Independence Measure version Ⅲ (SCIM Ⅲ). Nutritional status after SCI was assessed using the Prognostic Nutritional Index (PNI), Geriatric Nutritional Risk Index (GNRI), and Controlling Nutritional Status (CONUT) score. Recovery rates were calculated as the proportion of the maximum possible improvement achieved between 4 weeks and 6 months after SCI. Patients with higher PNI at 4 weeks after SCI showed greater neurological and functional recovery. Mean ISNCSCI total motor score recovery rates were 43.89%, 28.08%, and 18.7% in the good, normal, and poor PNI groups, respectively (p for trend = 0.0005), with corresponding SCIM III recovery rates of 40.65%, 26.1%, and 22.41% (p for trend = 0.0072). GNRI and CONUT scores were also associated with ISNCSCI total motor score recovery; however, PNI showed the strongest predictive performance (p = 0.0031). In multivariable linear regression analyses adjusting for age, sex, and baseline neurological and functional status, PNI remained independently associated with ISNCSCI total motor score recovery (β = 0.857 per 1-point increase, 95% CI 0.011–1.703, p = 0.0471). These findings indicate that early nutritional status, particularly as assessed by the PNI, is independently associated with neurological recovery after cervical SCI and may be useful for early prognostic assessment.

## Introduction

Cervical spinal cord injury (SCI) often results in severe motor and sensory deficits of the upper and lower limbs, leading to substantial impairment in activities of daily living (ADL) [1,2]. In addition to these functional limitations, patients with SCI are prone to systemic complications, including sepsis and malnutrition, which are associated with increased mortality [3]. Impaired swallowing function is common after cervical SCI, and dysphagia with subsequent aspiration pneumonia can further limit oral intake, thereby exacerbating malnutrition [4,5]. Moreover, neurological impairments after SCI, such as respiratory dysfunction, bladder and bowel dysfunction, and sensory loss, are associated with an increased risk of complications including pneumonia, pressure ulcers, and urinary tract infections [6,7]. These recurrent or persistent complications can promote chronic inflammation and contribute to hypoalbuminemia and protein-energy malnutrition.

A primary consequence of SCI is the rapid and profound skeletal muscle atrophy that occurs in paralyzed limbs due to the loss of neural innervation [8]. In severe SCI cases, muscle atrophy is particularly substantial. Malnutrition, especially protein deficiency, may hinder recovery of muscle mass and strength [9]. Recovery of neurological function and ADL after SCI requires not only maintenance of joint mobility but also restoration of muscle mass and contractile function [10]. However, the relationship between early nutritional status and subsequent neurological and functional recovery has not been fully elucidated.

Several standardized measures are commonly used to assess nutritional status in clinical practice, including the Prognostic Nutritional Index (PNI), the Geriatric Nutritional Risk Index (GNRI), and the Controlling Nutritional Status (CONUT) score. PNI combines serum albumin and total lymphocyte count, reflecting both protein nutritional status and immune function [11,12]. GNRI, based on serum albumin and the ratio of current to ideal body weight, was developed to assess nutritional risk and prognosis in older adults [13]. CONUT, which incorporates serum albumin, lymphocyte count, and total cholesterol, allows rapid nutritional screening without requiring anthropometric measurements and captures nutritional (protein-energy) as well as immunological status [14]. Each index therefore emphasizes different aspects of nutritional status, which may differentially relate to neurological and functional recovery after SCI.

The objective of this study was to investigate the impact of early nutritional status, as assessed by PNI, GNRI, and CONUT, on subsequent neurological function and ADL recovery in patients with cervical SCI. This study evaluated nutritional status, neurological function, and ADL performance at 4 weeks and 6 months after injury.

## Materials and methods

### Study design and participants

This study was a retrospective analysis of data obtained from a prospectively maintained cohort. We included patients with acute traumatic cervical SCI admitted to our center within 72 hours of injury between November 2016 and July 2022. Eligible patients were those with the potential for at least 6 months of follow-up. Exclusion criteria were absence of paralysis, early discharge or transfer to another hospital, history of stroke or dementia, or transfer due to systemic deterioration. A total of 206 patients met the initial inclusion criteria. Of these, 94 were excluded due to early discharge or transfer, 7 due to a history of stroke or dementia, and 14 due to systemic deterioration. In this study, systemic deterioration was defined as the occurrence of severe medical complications that necessitated transfer to other facilities for acute intensive management, such as severe respiratory or urinary tract infections, acute abdominal conditions, or sepsis. Consequently, 91 patients were included in the final cohort for the present analysis.

All participants received specialized inpatient rehabilitation at our spinal injury center. At the 4-week post-injury assessment, all patients were undergoing a comprehensive multidisciplinary rehabilitation program. The rehabilitation program followed a standardized institutional protocol, which is summarized in Supplemental Table 1. This program involved a systematic progression from acute stabilization and bedside therapy to comprehensive functional training, including mobility exercises and activities of ADL. Throughout the inpatient stay, patients received daily physical and occupational therapy tailored to the neurological level and severity of their injury to ensure a consistent approach to recovery.

Nutritional status was managed according to a standardized institutional protocol overseen by a Nutrition Support Team (NST). All patients underwent regular nutritional screening using the Malnutrition Universal Screening Tool (MUST) [15]. For patients identified as being at risk (MUST score ≥ 2), a formal diagnosis of malnutrition and its severity was performed based on the Global Leadership Initiative on Malnutrition (GLIM) criteria [16]. Phenotypic assessment included monitoring of unintentional weight loss, Body Mass Index (BMI), and skeletal muscle mass index (SMI) measured via bioelectrical impedance analysis. Etiologic assessment focused on reduced oral intake and the presence of systemic inflammation following acute trauma. Based on these evaluations, individualized nutritional support was provided, including protein-rich oral nutritional supplements or enteral nutrition, to address the hypermetabolic state associated with the subacute phase of SCI.

The study was approved by the Ethical Review Board of the Japan Labor Health and Welfare Organization Spinal Injuries Center (Approval code: 25−20). We confirm that this research was conducted in accordance with relevant guidelines and regulations, and it adhered to the principles outlined in the Declaration of Helsinki. We obtained all necessary consent from the patients and/or their legal guardians who participated in the study, including consent to participate in the study. Written informed consent for the publication of their clinical details was obtained for the patients.

### Clinical evaluation

Demographic and clinical data, including age, sex, and treatment strategy, were collected at baseline. Neurological status was assessed using the International Standards for Neurological Classification of Spinal Cord Injury (ISNCSCI) total motor score [17], and functional independence was evaluated using the Spinal Cord Independence Measure version Ⅲ (SCIM Ⅲ) [18,19]. The severity of SCI was classified using the American Spinal Injury Association Impairment Scale (AIS), where AIS A indicates complete injury, AIS B sensory incomplete injury, AIS C motor incomplete injury with less than half of key muscle functions below the single neurological level of injury (NLI) have a Manual Muscle Test (MMT) grade ≥ 3, and AIS D motor incomplete injury with at least half of the key muscles having an MMT grade ≥ 3 [20]. Assessments were performed at 4 weeks and 6 months after injury.

### Nutritional assessment

Nutritional status was evaluated at 4 weeks and 6 months using three established indices: the PNI, GNRI, and CONUT. The PNI, an indicator of nutritional and immunological status, was calculated as follows: PNI = 10 × serum albumin (g/dL) + 0.005 × total lymphocyte count (/mm^3^) [11,12]. Patients were categorized based on the PNI as follows: good (PNI ≥ 45), normal (40 ≤ PNI < 45), and poor (PNI < 40) nutritional status (S2 Table).

The GNRI, an indicator of nutritional risk, was calculated using the following formula: GNRI = (1.489 × serum albumin [g/L]) + (41.7 × present body weight / ideal body weight) [13]. Patients were categorized according to GNRI as follows: no nutritional risk (GNRI ≥ 98), low nutritional risk (92 ≤ GNRI < 98), moderate nutritional risk (82 ≤ GNRI < 92), and major nutritional risk (GNRI < 82) (S3 Table).

The CONUT score, an indicator of nutritional (protein-energy) and immunological status, was calculated based on serum albumin concentration, total lymphocyte count, and total cholesterol level. Each parameter was scored according to predefined cut-off values, and the sum of the three scores was used as the CONUT score. Patients were categorized according to the CONUT score as follows: normal nutritional status (CONUT ≤ 1), mild malnutrition (2 ≤ CONUT < 5), moderate malnutrition (5 ≤ CONUT < 9), and severe malnutrition (CONUT ≥ 9) (S4 and S5 Tables) [14].

Nutritional status was evaluated at 4 weeks and 6 months after injury. The 4-week time point was selected as the baseline for early nutritional status because biochemical markers such as serum albumin are strongly influenced by the acute inflammatory response and surgical stress during the hyper-acute phase after spinal cord injury [21,22]. Consequently, assessments performed immediately after injury may primarily reflect physiological stress responses rather than the patient’s underlying nutritional condition. Previous studies have demonstrated that the prognostic value of serum albumin measurements increases progressively over the first month after injury, with 4-week assessments showing stronger associations with long-term neurological outcomes than earlier measurements [23]. In addition, by 4 weeks post-injury, most patients have completed acute medical stabilization and entered the intensive rehabilitation phase, providing a more stable baseline for prognostic evaluation.

### Outcome measures and recovery rates

Neurological recovery was assessed using the ISNCSCI total motor score, and functional recovery was evaluated using SCIM III. The improvement rate of the ISNCSCI total motor score was calculated as the difference between the 6-month score and the 4-week score divided by the possible remaining improvement [24], expressed as a percentage: Motor score recovery rate (%) = (ISNCSCI total motor score at 6 months − ISNCSCI total motor score at 4 weeks) ÷ (100 − ISNCSCI total motor score at 4 weeks) × 100. Similarly, the SCIM III improvement rate was calculated as the difference between the 6-month score and the 4-week score divided by the possible remaining improvement, expressed as a percentage: SCIM III recovery rate (%) = (SCIM III score at 6 months − SCIM III score at 4 weeks) ÷ (100 − SCIM III score at 4 weeks) × 100.

### Statistical analysis

Continuous variables are presented as mean ± standard deviation (SD), and categorical variables are presented as counts and percentages. To examine the associations between recovery rates and nutritional status categories at 4 weeks after SCI, linear trend analyses were performed across the ordered categories of each nutritional index (PNI, GNRI, and CONUT). Nutritional categories were treated as ordinal variables, and trend effects were evaluated using linear regression models with recovery rate of ISNCSCI total motor score or SCIM III score as dependent variables. To further assess the relationships between nutritional indices measured at 4 weeks and recovery outcomes, correlation analyses were conducted using simple linear regression analyses based on Pearson’s correlation coefficients. The recovery rate of ISNCSCI total motor score and the recovery rate of SCIM III score were used as outcome variables, and the coefficients of determination (R^2^) and corresponding P values were reported. Multivariable linear regression analyses were performed to identify independent factors associated with neurological recovery and functional recovery. The recovery rate of ISNCSCI total motor score was used as an indicator of neurological recovery, and the recovery rate of SCIM III score was used as an indicator of functional recovery. Age, sex, nutritional status at 4 weeks (PNI), ISNCSCI total motor score at 4 weeks, and SCIM III score at 4 weeks were included as covariates in the multivariable models based on clinical relevance. Regression coefficients (β), 95% confidence intervals (CI), and P values were calculated. Model fit was assessed using the coefficient of determination (R^2^). All statistical tests were two-sided, and statistical significance was defined as P < 0.05. All statistical analyses were performed using JMP software (version 14; SAS Institute Inc., Cary, NC, USA).

## Results

### Study demographics and clinical characteristics

A total of 91 patients with cervical SCI were included in this study. The mean age was 60.8 ± 16.63 years (range, 17–83 years), and 74 patients (81.32%) were male. Regarding treatment strategy, 44 patients (48.35%) underwent surgical treatment, whereas 47 patients (51.65%) were managed nonoperatively. The mean height and body weight were 165.4 ± 8.31 cm (range, 145–182 cm) and 60.96 ± 11.19 kg (range, 34.5–89 kg), respectively, with a mean body mass index (BMI) of 22.18 ± 3.21 (range, 15.33–30.09). The mean length of hospital stay was 322.95 ± 90.2 days (range, 182–591 days). Regarding comorbidities, 17 patients (18.68%) had diabetes mellitus; however, all were clinically stable and received appropriate medical management throughout their hospitalization. No other specific metabolic or endocrine disorders, such as thyroid dysfunction or chronic hepatic disease, were identified in our cohort. Baseline renal function assessment showed that 73 patients (80.22%) had an estimated glomerular filtration rate (eGFR) ≥ 60 mL/min/1.73 m^2^, while 18 patients (19.78%) had an eGFR between 30 and 60 mL/min/1.73 m^2^; no patients had an eGFR < 30 mL/min/1.73 m^2^. At 4 weeks after SCI, the distribution of the AIS grades was as follows: AIS A in 25 patients (27.47%), AIS B in 15 patients, AIS C in 21 patients, and AIS D in 30 patients. The neurological level of injury (NLI) was most frequently observed at the C4 level, accounting for 46 patients (50.5%), with the remaining patients distributed across other cervical levels (S1 Fig). The mean ISNCSCI total motor score at 4 weeks was 36.53 ± 26 (range, 0–90), which improved to 49.41 ± 31.58 (range, 0–98) at 6 months after injury. The mean change in ISNCSCI total motor score was 12.88 ± 12.57 (range, −19–52), corresponding to a mean motor score recovery rate of 27.67 ± 27.81% (range, −36.54% – 88.46%). The mean SCIM III score at 4 weeks after injury was 18.09 ± 10.57 (range, 0–60), which increased to 36.69 ± 23.38 (range, 6–98) at 6 months. The mean change in SCIM III score was 21.6 ± 18.22 (range, −2–80), with a mean SCIM III recovery rate of 27.96 ± 24.84% (range, −2.38% – 95.74%) (Table 1). Regarding changes in neurological severity, only two of the 91 patients showed deterioration in the AIS grade at 6 months after injury, whereas the remaining 89 patients exhibited either no change or improvement in AIS grade during the follow-up period (S6 Table).

**Table 1:** Demographics of patients included in this study.

Demographics (n = 91)	
Age (years)	60.8 ± 16.63 (17 - 83)
Sex (M/F: cases)	74 (81.32%)/17
Treatment strategy for SCI (Operative/Nonoperative)	44 (48.35%)/47
Height (cm)	165.4 ± 8.31 (145 - 182)
Weight (kg)	60.96 ± 11.19 (34.5 - 89)
BMI	22.18 ± 3.21 (15.33 - 30.09)
Length of stay (days)	322.95 ± 90.2 (182 - 591)
Diabetes mellitus (yes/no)	17 (18.68%)/74
eGFR (mL/min/1.73 m^2^) (30–60/ ≥ 60)	18 (19.78%)/73
ASIA Impairment Scale at 4 weeks after SCI (A/B/C/D)	25 (27.47%)/15/21/30
ISNCSCI total motor score at 4 weeks after SCI	36.53 ± 26 (0 - 90)
ISNCSCI total motor score at 6 months after SCI	49.41 ± 31.58 (0 - 98)
Change in motor score	12.88 ± 12.57 (−19 - 52)
Recovery rate of motor score	27.67 ± 27.81 (−36.54–88.46)
SCIM III score at 4 weeks after SCI	18.09 ± 10.57 (0 - 60)
SCIM III score at 6 months after SCI	36.69 ± 23.38 (6 - 98)
Change in SCIM III score	21.6 ± 18.22 (−2 - 80)
Recovery rate of SCIM III score	27.96 ± 24.84 (−2.38 - 95.74)

SCI: Spinal Cord Injury; BMI: Body Mass Index; eGFR: estimated glomerular filtration rate; ASIA; American Spinal Injury Association; ISNCSCI: International Standards for Neurological Classification of Spinal Cord Injury; SCIM: Spinal Cord Independence Measure

Variables are given as the mean and standard deviation with the range in parenthesis or as the number with the percentage in parenthesis.

### Nutritional status at 4 weeks and 6 months after SCI

At 4 weeks after SCI, the mean serum albumin level was 3.356 ± 0.488 g/dL, total lymphocyte count was 1419 ± 440 /mm^3^, and total cholesterol level was 159.2 ± 31.9 mg/dL. The mean PNI score at 4 weeks was 40.67 ± 5.94, with 22 patients (24.18%) classified as good, 28 (30.77%) as normal, and 41 (45.05%) as poor nutritional status. The mean CONUT score at 4 weeks was 3.58 ± 2.55. According to CONUT categories, 24 patients (26.37%) were classified as normal, 33 (36.26%) as mild malnutrition, 30 (32.97%) as moderate malnutrition, and 4 (4.40%) as severe malnutrition. The mean GNRI score at 4 weeks was 89.5 ± 8.17, with 17 patients (18.68%) categorized as no risk, 20 (21.98%) as low risk, 37 (40.66%) as moderate risk, and 17 (18.68%) as severe risk. At 6 months after injury, nutritional parameters showed overall improvement. Serum albumin increased to 3.73 ± 0.449 g/dL, total lymphocyte count to 1674 ± 579 /mm^3^, and total cholesterol to 171 ± 34.3 mg/dL. The mean PNI score increased to 45.68 ± 6.06, with 52 patients (57.14%) classified as good, 21 (23.08%) as normal, and 18 (19.78%) as poor nutritional status. Similarly, the mean CONUT score decreased to 2.13 ± 2.03 at 6 months. Based on CONUT categories, 46 patients (50.55%) were classified as normal, 32 (35.16%) as mild malnutrition, 13 (14.29%) as moderate malnutrition, and no patients were classified as severe malnutrition. The mean GNRI score at 6 months was 95.06 ± 7.41, with 31 patients (34.07%) categorized as no risk, 32 (35.16%) as low risk, 24 (26.37%) as moderate risk, and 4 (4.40%) as severe risk (Table 2).

**Table 2:** Results of nutritional status in the study population.

Variables	4 weeks after SCI	6 months after SCI
Serum albumin (g/dL)	3.357 ± 0.488	3.73 ± 0.449
Total lymphocyte count (/mm³)	1419 ± 440	1674 ± 579
Total cholesterol (mg/dL)	159.2 ± 31.9	171 ± 34.3
PNI score	40.67 ± 5.94	45.68 ± 6.06
PNI categories (Good/Normal/Poor)	22 (24.18%)/28/41	52 (57.14%)/21/18
CONUT score	3.58 ± 2.55	2.13 ± 2.03
CONUT categories (Normal/Mild malnutrition/Moderate malnutrition/Severe malnutrition)	24 (26.37%)/33/30/4	46 (50.55%)/32/13/0
GNRI score	89.5 ± 8.17	95.06 ± 7.41
GNRI categories (No risk/Low risk/Moderate risk/Severe risk)	17 (18.68%)/20/37/17	31 (34.07%)/32/24/4

SCI: Spinal Cord Injury; PNI: Prognostic Nutritional Index; GNRI: Geriatric Nutritional Risk Index; CONUT: Controlling Nutritional Status

Variables are given as the mean and standard deviation with the range in parenthesis or as the number with the percentage in parenthesis.

### Changes in nutritional status between 4 weeks and 6 months after SCI

Detailed longitudinal shifts in nutritional status for each index are summarized in S7-S9 Tables. Overall, nutritional status improved in a substantial proportion of patients across all three indices, while deterioration was observed in only a minority of cases.

PNI: Among patients classified as having good nutritional status at 4 weeks, 90.91% (20/22) remained in the same category at 6 months. Of those classified as normal at 4 weeks, 71.43% (20/28) improved to the good category at 6 months, whereas only 10.71% (3/28) deteriorated to the poor category. Notably, 63.41% (26/41) of patients in the poor group at 4 weeks showed improvement at 6 months, with 29.27% (n = 12) reaching the good category and 34.15% (n = 14) moving to normal.

GNRI: Regarding nutritional risk, 82.35% (14/17) of the no risk group at 4 weeks remained stable at 6 months. Among patients at moderate risk at 4 weeks, 59.46% (22/37) improved to either the no-risk or low-risk category at 6 months, while only 5.4% (2/37) progressed to severe risk. Additionally, 88.24% (15/17) of those initially at severe risk improved to the low-risk or moderate-risk category at 6 months.

CONUT score: Similar positive trends were observed for the CONUT score. Of the patients with mild malnutrition at 4 weeks, 60.61% (20/33) improved to the normal category at 6 months. Furthermore, 70% (21/30) of patients with moderate malnutrition at 4 weeks improved to either normal or mild malnutrition at 6 months. Importantly, no patients were classified as having severe malnutrition at 6 months.

### Linear trends between nutritional status categories and recovery rates

Linear trend analyses were performed to examine the associations between nutritional status categories at 4 weeks after SCI and subsequent recovery rates of ISNCSCI total motor score and SCIM III (Table 3). Nutritional status categories were treated as ordinal variables, and β coefficients represent the change in recovery rate per one-category worsening of nutritional status. For PNI categories, recovery rates of both ISNCSCI total motor score and SCIM III decreased progressively with worsening nutritional status. The mean recovery rate of ISNCSCI total motor score was 43.89 ± 6.29% in the good group, 28.08 ± 5.02% in the normal group, and 18.7 ± 3.76% in the poor group. A significant negative linear trend was observed, with a decrease of 12.278 percentage points per one-category worsening (β = −12.278, 95% CI −19.031 to −5.525; p for trend = 0.0005). Similarly, the SCIM III recovery rate declined from 40.65 ± 5.5% in the good group to 26.1 ± 4.83% in the normal group and 22.41 ± 3.4% in the poor group, demonstrating a significant linear trend (β = −8.582, 95% CI −14.784 to −2.381; p for trend = 0.0072). For GNRI categories, worsening nutritional risk was also associated with lower ISNCSCI total motor score recovery rates. The mean recovery rates were 39.96 ± 6.93% in the no-risk group, 33.23 ± 7.51% in the low-risk group, 23.92 ± 3.57% in the moderate-risk group, and 17.02 ± 6.76% in the severe-risk group. A significant negative linear trend was identified, with a decrease of 7.89 percentage points per one-category increase in GNRI risk (β = −7.893, 95% CI −13.51 to −2.277; p for trend = 0.0064). In contrast, although SCIM III recovery rates tended to decrease with worsening GNRI category, the linear trend did not reach statistical significance (β = −4.738, 95% CI −9.875 to 0.399; p for trend = 0.0702). For CONUT categories, ISNCSCI total motor score recovery rates decreased with increasing severity of malnutrition, from 42.27 ± 6.81% in the normal group to 22.72 ± 4.22% in the mild malnutrition group and 21.23 ± 4.09% in the moderate malnutrition group. A significant negative linear trend was observed (β = −7.828, 95% CI −14.369 to −1.288; p for trend = 0.0195). Similarly, SCIM III recovery rates declined across CONUT categories, with a significant linear trend indicating a decrease of 7.343 percentage points per one-category worsening of malnutrition severity (β = −7.343, 95% CI −13.167 to −1.52; p for trend = 0.014).

**Table 3:** Linear trends in ISNCSCI total motor score and SCIM III improvement rates across nutritional status categories.

	PNI categories at 4weeks after SCI						
Variables	Good		Normal	Poor	β (per category)	95% CI	P for trend
Recovery rate of ISNCSCI total motor score	43.89 ± 6.29		28.08 ± 5.02	18.7 ± 3.76	−12.278	−19.031 to −5.525	0.0005
Recovery rate of SCIM III score	40.65 ± 5.5		26.1 ± 4.83	22.41 ± 3.4	−8.582	−14.784 to −2.381	0.0072
	**GNRI categories at 4weeks after SCI**						
**Variables**	**No risk**	**Low risk**	**Moderate risk**	**Severe risk**	**β (per category)**	**95% CI**	**P for trend**
Recovery rate of ISNCSCI total motor score	39.96 ± 6.93	33.23 ± 7.51	23.92 ± 3.57	17.02 ± 6.76	−7.893	−13.51 to −2.277	0.0064
Recovery rate of SCIM III score	37.21 ± 6.07	31.81 ± 6.42	22.55 ± 3.18	25.93 ± 7.03	−4.738	−9.875 to 0.399	0.0702
	**CONUT categories at 4weeks after SCI**						
**Variables**	**Normal**	**Mild malnutrition**	**Moderate malnutrition**	**Severe malnutrition**	**β (per category)**	**95% CI**	**P for trend**
Recovery rate of ISNCSCI total motor score	42.27 ± 6.81	22.72 ± 4.22	21.23 ± 4.09	29.24 ± 16.83	−7.828	−14.369 to −1.288	0.0195
Recovery rate of SCIM III score	39.04 ± 5.87	25.63 ± 3.92	23.08 ± 3.93	17.25 ± 14.63	−7.343	−13.167 to −1.52	0.014

ISNCSCI: International Standards for Neurological Classification of Spinal Cord Injury; SCIM: Spinal Cord Independence Measure; SCI: Spinal Cord Injury; PNI: Prognostic Nutritional Index; GNRI: Geriatric Nutritional Risk Index; CONUT: Controlling Nutritional Status; CI: Confidence Interval

Variables are given as the mean and standard deviation.

### Correlation analyses between nutritional indices and recovery rates

**Fig 1:**
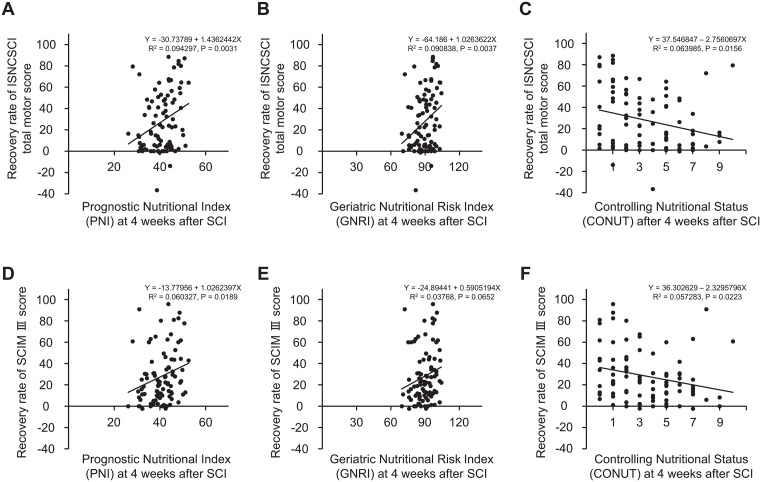
Correlation analyses between nutritional indices and neurological and functional recovery after cervical spinal cord injury (SCI). Linear regression analyses using Pearson’s correlation coefficients were performed to evaluate the relationships between nutritional indices measured at 4 weeks after SCI and recovery outcomes, including the recovery rates of International Standards for Neurological Classification of Spinal Cord Injury (ISNCSCI) total motor score and Spinal Cord Independence Measure (SCIM) III score. (A) Correlation between the Prognostic Nutritional Index (PNI) and recovery rate of ISNCSCI total motor score (R^2^ = 0.094297, P = 0.0031). (B) Correlation between the Geriatric Nutritional Risk Index (GNRI) and recovery rate of ISNCSCI total motor score (R^2^ = 0.090838, P = 0.0037). (C) Correlation between the Controlling Nutritional Status (CONUT) score and recovery rate of ISNCSCI total motor score (R^2^ = 0.063985, P = 0.0156). (D) Correlation between PNI and recovery rate of SCIM III score (R^2^ = 0.060327, P = 0.0189). (E) Correlation between GNRI and recovery rate of SCIM III score (R^2^ = 0.03768, P = 0.0652). (F) Correlation between CONUT score and recovery rate of SCIM III score (R^2^ = 0.057283, P = 0.0223).

Based on the observed differences in recovery rates across nutritional status categories, correlation analyses were performed to evaluate whether nutritional indices measured at 4 weeks after SCI were associated with neurological and functional recovery outcomes when treated as continuous variables (Fig 1). Linear regression analyses using Pearson’s correlation coefficients demonstrated significant correlations between nutritional indices and the recovery rate of ISNCSCI total motor score. The PNI showed a positive correlation with ISNCSCI total motor score recovery rate (R^2^ = 0.094297, p = 0.0031; Fig 1A). Similar but slightly weaker correlations were observed for the GNRI (R^2^ = 0.090838, p = 0.0037; Fig 1B) and the CONUT score (R^2^ = 0.063985, p = 0.0156; Fig 1C). Regarding functional recovery, PNI was also significantly correlated with the recovery rate of SCIM III score (R^2^ = 0.060327, p = 0.0189; Fig 1D). The CONUT score demonstrated a significant correlation with SCIM III recovery rate as well (R^2^ = 0.057283, p = 0.0223; Fig 1F), whereas the correlation between GNRI and SCIM III recovery rate did not reach statistical significance (R^2^ = 0.03768, p = 0.0652; Fig 1E). Although the coefficients of determination were modest across all analyses, indicating that nutritional indices alone explained a limited proportion of the variance in recovery rates, the consistent statistical significance observed, especially for PNI, suggests a meaningful association between early nutritional status and subsequent neurological and functional recovery. Taken together with the results of the trend analyses across nutritional categories, these findings indicate that among the three nutritional indices evaluated, PNI may be the most informative indicator for predicting recovery after cervical SCI.

### Multivariable linear regression analyses for neurological and functional recovery

Multivariable linear regression analyses were conducted to identify independent predictors of recovery, with the models for neurological recovery (ISNCSCI total motor score improvement rate) and functional recovery (SCIM III recovery rate) summarized in Table 4. Both models included age, sex, and 4-week baseline values for PNI, ISNCSCI total motor score, and SCIM III score as covariates.

**Table 4:** Multivariable linear regression analysis of factors associated with neurological and functional recovery.

	Neurological recovery (ISNCSCI total motor score improvement rate)			Functional recovery (SCIM III improvement rate)		
Variables	β	95% CI	P value	β	95% CI	P value
Age (per 1 year)	−0.008	−0.34 to 0.323	0.9599	−0.275	−0.531 to −0.018	0.0362
Sex (female vs male)	−3.971	−9.661 to 1.718	0.1688	−0.536	−4.941 to 3.869	0.8094
PNI at 4 weeks (per 1 point)	0.857	0.011 to 1.703	0.0471	−0.02	−0.676 to 0.634	0.9503
ISNCSCI total motor score at 4 weeks (per 1 point)	0.724	0.488 to 0.961	< 0.0001	0.767	0.584 to 0.95	< 0.0001
SCIM III score at 4 weeks (per 1 point)	−0.246	−0.797 to 0.304	0.3764	0.068	−0.359 to 0.494	0.7526

ISNCSCI: International Standards for Neurological Classification of Spinal Cord Injury; SCIM: Spinal Cord Independence Measure; PNI: Prognostic Nutritional Index; CI: Confidence Interval.

For neurological recovery, the model explained 47.54% of the variance (R^2^ = 0.4754). After adjusting for potential confounders, PNI at 4 weeks remained independently associated with the total motor score improvement rate (β = 0.857 per 1-point increase, 95% CI 0.011–1.703; p = 0.0471). Additionally, a higher baseline motor score was strongly associated with greater improvement (β = 0.724 per 1-point increase, 95% CI 0.488–0.961; p < 0.0001). In this model, age, sex, and baseline SCIM III score did not show independent associations.

For functional recovery, the model showed that PNI at 4 weeks was not an independent predictor of SCIM III improvement (β = −0.02, 95% CI −0.676 to 0.634; p = 0.9503). Instead, older age was significantly associated with a lower recovery rate (β = −0.275 per year, 95% CI −0.531 to −0.018; p = 0.0362), while a higher baseline motor score was independently associated with greater functional gains (β = 0.767 per 1-point increase, 95% CI 0.584–0.950; p < 0.0001). Sex and baseline SCIM III score showed no significant independent associations with the outcome.

## Discussion

### Early nutritional status as a predictor of neurological recovery

This study investigated the prognostic role of nutritional status on neurological and functional recovery in patients with cervical SCI. The key finding is that nutritional status at 4 weeks post-injury, particularly as assessed by the PNI, was an independent predictor of subsequent neurological recovery, measured by improvement in the ISNCSCI total motor score. This association between nutritional status and neurological recovery remained significant after adjusting for age, sex, and baseline neurological and functional status. Our findings highlight the clinical importance of early nutritional management after SCI. Although nutritional status was not an independent predictor of functional recovery in multivariable analysis, it was significantly associated in univariate and correlation analyses. These findings suggest that nutritional status contributes to functional recovery indirectly, likely through its influence on neurological improvement. Ultimately, functional recovery, as measured by the SCIM III, is determined by a complex interplay of multiple factors, such as the extent of neurological improvement, age, and rehabilitation intensity, and not by nutritional status alone.

### Biological rationale for the predictive strength of PNI

The predictive strength of PNI can be attributed to its components, consisting of serum albumin and total lymphocyte count, which effectively capture key aspects of the complex pathophysiology following cervical SCI. Serum albumin is not only a marker of visceral protein status but also a major acute-phase protein, and its serum concentration decreases when systemic inflammation is present [21,25]. Following cervical SCI, patients are highly susceptible to secondary complications such as pneumonia and urinary tract infections, which trigger a significant inflammatory response [1]. Inflammatory cytokines, including interleukin-6 and tumor necrosis factor-α, increase vascular permeability and facilitate the movement of albumin into the extravascular space [26]. Furthermore, inflammation modifies hepatic protein synthesis, leading to decreased albumin synthesis and increased production of positive acute-phase proteins, such as C-reactive protein [27]. In addition, the total lymphocyte count reflects immune function and nutritional status, as a reduction in lymphocyte count is commonly observed in conditions of physiological stress and malnutrition [28,29]. Therefore, the PNI represents a clinically meaningful index that captures both protein-energy malnutrition and inflammation-related immune suppression. This combined assessment may reflect the patient’s capacity for tissue repair, preservation of skeletal muscle mass, and resistance to infection, all of which are essential for neurological recovery.

### Prognostic performance of PNI compared with GNRI and CONUT

In this study, PNI demonstrated superior and more consistent predictive performance compared with GNRI and CONUT. The difference observed with GNRI can be explained by its original purpose and components. GNRI was initially developed to evaluate nutritional risk and prognosis in older populations and includes body weight in its calculation [13]. As the population of patients with SCI is progressively aging [30,31], GNRI may become increasingly relevant for nutritional assessment in this group. However, in the subacute phase following SCI, rapid muscle atrophy due to paralysis and substantial changes in body fluid distribution limit the ability of body weight to reflect nutritional status accurately [32]. In our cohort, the direct biochemical markers of PNI may have provided a more stable and relevant assessment. However, given the trend of increasing age at injury, GNRI may become more pertinent for risk stratification in future SCI cohorts. The CONUT score, while also useful, was a slightly weaker predictor than PNI. The primary difference is CONUT’s inclusion of total cholesterol. Cholesterol levels can be highly variable in the subacute phase, influenced by inconsistent dietary intake, metabolic stress [33], and its role as a negative acute-phase reactant during inflammation, potentially rendering it a less reliable indicator of recovery potential compared to the more direct markers of protein stores and immune function captured by the PNI.

### Challenges of PNI as a nutritional assessment tool

The prognostic value of PNI likely stems from its ability to reflect both protein-energy status and the systemic inflammatory burden. It is well-established that serum albumin is a negative acute-phase reactant, and its levels decrease in response to systemic inflammation regardless of nutritional intake [34,35]. Patients with cervical SCI are particularly susceptible to secondary complications, such as pneumonia, urinary tract infections (UTIs), and pressure ulcers, all of which trigger a persistent inflammatory state [36,37]. Consequently, a low PNI in the subacute phase may function as a surrogate marker for a high complication burden, which hinders neurological recovery. While PNI remains a useful clinical tool, our findings highlight the need for more objective and comprehensive nutritional assessment methods, such as bioelectrical impedance analysis or muscle ultrasound, that can distinguish between primary malnutrition and inflammation-induced metabolic changes.

### Nutritional support for neural repair and muscle preservation

The biological mechanisms linking enhanced nutritional status to neurological recovery are likely multifactorial. Optimal protein and energy intake is essential for attenuating the catabolic state that develops after SCI [38]. Sufficient nutritional support provides substrates necessary for regulating secondary injury processes, including excessive neuroinflammation, and for supporting endogenous repair mechanisms. Axonal sprouting, remyelination, and synaptic plasticity are energy-dependent processes that require continuous availability of amino acids, lipids, and micronutrients [39]. In addition, adequate nutritional status helps to mitigate severe denervation-induced skeletal muscle atrophy [9]. Preservation of muscle mass is critical, as skeletal muscle is required to convert restored neural signals into functional movement. Conversely, malnutrition may impair neural repair and accelerate muscle loss, thereby limiting the potential for neurological and functional recovery. In addition to the total intake, the specific route of nutritional support, such as oral, enteral, or parenteral delivery, can also influence the systemic inflammatory environment. At our institution, patients were managed according to a standardized protocol by a Nutrition Support Team, which prioritizes early enteral or oral nutrition to maintain gut integrity and modulate inflammation. However, since specific data on feeding routes and daily dosages for each patient were not collected in this retrospective study, we could not analyze these factors as potential confounding variables. Given that different nutritional strategies can affect both the biochemical markers in the PNI and the recovery rates, this remains an important area for future prospective investigation.

### Neurological recovery as a foundation for functional independence

A key finding of this study was the divergent impact of nutritional status on neurological versus functional outcomes. Specifically, the PNI independently predicted improvements in the ISNCSCI total motor score but not in the SCIM III score. This discrepancy is consistent with previous reports demonstrating that neurological recovery does not necessarily translate directly into functional independence after SCI [40,41]. Achieving functional independence is a complex, multifactorial process. While neurological improvement constitutes the essential biological substrate for recovery, the ability to perform ADL is strongly influenced by non-neurological factors, including age, rehabilitation intensity, psychosocial support, and the management of secondary complications such as spasticity and pain [42]. Indeed, SCIM III was specifically developed to capture functional outcomes that extend beyond neurological impairment and is therefore particularly sensitive to these contextual and environmental factors [18]. In line with this concept, our multivariable model for SCIM III recovery identified age and baseline motor function as the primary predictors, a finding that is well supported by prior studies [43,44]. Taken together, these results suggest that nutritional status functions as a foundational determinant that enhances the potential for neurological repair, thereby creating a prerequisite for functional gains that can only be fully realized through comprehensive and multidisciplinary rehabilitation.

### Clinical implications and future directions

Our findings in this study have important clinical implications. Serial assessment of the PNI during the subacute phase of cervical SCI may provide a simple and cost-effective approach to identify patients at high risk for poor neurological recovery. Malnutrition is common after SCI, largely driven by hypermetabolism and systemic inflammation, and has been associated with adverse clinical outcomes [38,45]. For patients with a low or declining PNI, clinicians may be justified in implementing more aggressive nutritional strategies, including early initiation of enteral nutrition, the use of high-protein formulations, or supplementation with specific amino acids and immunonutrients, all of which have been shown to mitigate catabolism and support tissue repair in critically ill and neurologically injured populations [46,47]. These results support the concept that nutritional optimization should not be regarded as an ancillary aspect of care, but rather as a primary therapeutic target to maximize neurological recovery potential after SCI [48]. Notably, the initial four weeks following injury represent a period of profound metabolic instability and heightened vulnerability to nutritional deterioration. Therefore, this early post-injury period may represent a critical therapeutic window in which nutritional interventions are likely to be most effective. Future studies should focus on longitudinally characterizing nutritional markers beginning at hospital admission, as dynamic changes in PNI during this early phase may provide greater prognostic value than a single time-point assessment and help refine individualized nutritional strategies for patients with SCI.

### Limitations of the study

This study has several limitations. First, as an observational study, we cannot establish causality; a low PNI may be a marker of injury severity and complications rather than a direct cause of poor recovery. Specifically, a low PNI might represent a persistent inflammatory state associated with secondary complications such as pneumonia or pressure ulcers, which are common after cervical SCI. Second, a significant selection bias may exist because we excluded 94 patients who were discharged or transferred before the 6-month follow-up. These excluded patients often had milder injuries and better initial recovery, allowing for earlier discharge. As a result, our final cohort of 91 patients, who had an average length of stay of over 300 days, represents a more severe subpopulation of cervical SCI requiring intensive, long-term inpatient care. While this may limit the generalizability of our results to patients with milder SCI, the findings remain highly relevant for the management of patients with severe injuries who are at the highest risk for malnutrition and long-term disability. Third, while BMI was recorded at baseline, it has known limitations in the SCI population. BMI may not accurately reflect actual body composition or cardiometabolic risk in these patients, as the rapid muscle atrophy and changes in fat distribution following paralysis can make BMI a less reliable indicator of nutritional status compared to other measures. Fourth, we did not collect detailed data on the specific nutritional interventions, such as precise caloric and protein intake, for each patient throughout the follow-up period. Consequently, we were unable to include nutritional management as a controlled variable in our analysis to evaluate its direct impact. Future prospective studies incorporating these management details are needed to clarify the relationship between specific nutritional support and recovery. In addition, we did not analyze individual data regarding the specific mode of nutritional delivery (oral, enteral, or total parenteral nutrition) or the exact caloric and protein dosages provided during the initial post-injury weeks. Because the route and content of nutrition can significantly influence inflammatory markers and metabolic recovery, the lack of these detailed records remains a confounding factor in our analysis. Finally, a larger, multicenter prospective study is necessary to validate these findings. A randomized controlled trial is ultimately warranted to investigate whether therapeutic strategies aimed at directly improving the PNI score can causally enhance neurological recovery and functional independence.

## Conclusions

In patients with cervical SCI, nutritional status assessed at 4 weeks after injury was significantly associated with subsequent recovery outcomes. Among the indices evaluated, the PNI emerged as the most informative marker, showing a consistent and independent association with neurological recovery as measured by the ISNCSCI total motor score improvement rate. These findings highlight the importance of early nutritional status in the recovery process and suggest that nutritional assessment and optimization should be integrated into the comprehensive care of patients after cervical SCI to maximize their recovery potential.

**Key Points**

- The authors evaluated the relationship between nutritional status and neurological and functional recovery in 91 prospectively registered cervical SCI patients.
- Nutritional status at 4 weeks and 6 months was assessed using PNI, GNRI, and CONUT scores.
- Worsening nutritional status was associated with lower recovery rates of ISNCSCI total motor score and SCIM III score.
- PNI was independently predictive of neurological recovery based on ISNCSCI total motor score.
- Early nutritional assessment and optimization may improve neurological and functional outcomes.

## Supporting information

S1 Fig Neurological level of injury (NLI) in the study population. (A) The distribution of the NLI among the study participants is shown. The most common NLI was C4, observed in 46 patients (50.5%).
(PDF)

S1 Table Institutional standardized rehabilitation protocol for cervical spinal cord injury. (DOCX)

S2 Table Classification of nutritional status based on prognostic nutritional index. (DOCX)

S3 Table Classification of nutritional status based on geriatric nutritional risk index. (DOCX)

S4 Table Controlling nutritional status (CONUT) score. (DOCX)

S5 Table Classification of nutritional status based on controlling nutritional status. (DOCX)

S6 Table Changes in ASIA impairment scale at 4 weeks and 6 months after SCI. (DOCX)

S7 Table Changes in prognostic nutritional index categories at 4 weeks and 6 months after SCI. (DOCX)

S8 Table Changes in geriatric nutritional risk index categories at 4 weeks and 6 months after SCI. (DOCX)

S9 Table Changes in controlling nutritional status categories at 4 weeks and 6 months after SCI. (DOCX)

S1 File Human participants research checklist. (DOCX)

## Abbreviations

ADL: Activities of Daily Living
ASIA: American Spinal Injury Association
AIS: ASIA Impairment Scale
BMI: Body Mass Index
CONUT: Controlling Nutritional Status
GNRI: Geriatric Nutritional Risk Index
ISNCSCI: International Standards for Neurological Classification of Spinal Cord Injury
MMT: Manual Muscle Test
NLI: Neurological Level of Injury
PNI: Prognostic Nutritional Index
SCI: Spinal Cord Injury
SCIM: Spinal Cord Independence Measure
