# Supplementary figures and images for: Prognostic significance of nutritional status for neurological and functional recovery after cervical spinal cord injury
Source: PLoS One. 2026 Jul 7;21(7):e0353302. doi: 10.1371/journal.pone.0353302 (PMC13340789; doi:10.1371/journal.pone.0353302)

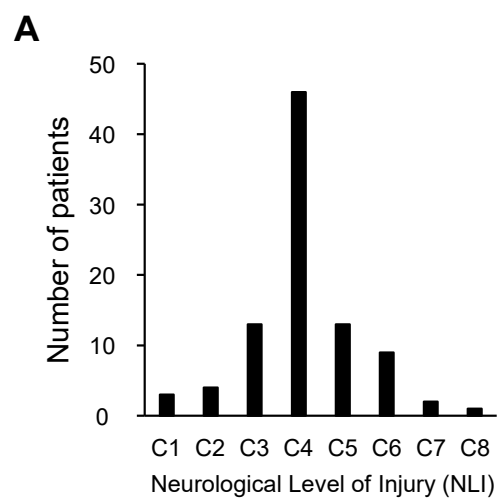

Supplemental figure 1

Supplement: S1 Fig — (A) The distribution of the NLI among the study participants is shown. The most common NLI was C4, observed in 46 patients (50.5%). (PDF) [file pone.0353302.s001.pdf]
