# Supplementary material for: Prognostic significance of nutritional status for neurological and functional recovery after cervical spinal cord injury
Source: PLoS One. 2026 Jul 7;21(7):e0353302. doi: 10.1371/journal.pone.0353302 (PMC13340789; doi:10.1371/journal.pone.0353302)
Supplement: S1 Table — (DOCX) [file pone.0353302.s002.docx]

**Supplemental Table 1. Institutional Standardized Rehabilitation Protocol for Cervical Spinal Cord Injury**

| Phase | Timeline | Physical Therapy (PT) | Occupational Therapy (OT) |
| --- | --- | --- | --- |
| Acute Phase | 1–7 days | Prevention of complications (respiratory, skin), Passive Range of Motion (ROM), Initiation of upright sitting tolerance (Bed-up). | Splinting for upper extremity positioning, Basic ADL assessment (e.g., eating). |
| Early Subacute Phase | 1–2 weeks | Increasing upright sitting tolerance (Bed-up full), Initiation of wheelchair sitting, Sitting balance training. | Training for basic self-care (grooming, facial washing), Assessment of assistive devices for feeding. |
| Transition Phase (Assessment Point) | 3–4 weeks | Transfer training (use of sliding board), Wheelchair mobility, Muscle strengthening of non-paralyzed muscles. | Intensive ADL training (eating, dressing), Selection and adjustment of wheelchairs and assistive devices. |
| Comprehensive Rehab Phase | 1–6 months | Advanced transfer training, Standing practice (tilt table), Gait training (if applicable), Aerobic exercise (arm-crank). | Social reintegration training, Home environment evaluation, Advanced ADL training (toileting, bathing). |
| Discharge Preparation | >6 months | Final assessment of mobility, Community reintegration programs. | Planning for home modification and social/vocational support. |
