# Supplementary material for: Prognostic significance of nutritional status for neurological and functional recovery after cervical spinal cord injury
Source: PLoS One. 2026 Jul 7;21(7):e0353302. doi: 10.1371/journal.pone.0353302 (PMC13340789; doi:10.1371/journal.pone.0353302)
Supplement: S2 Table — (DOCX) [file pone.0353302.s003.docx]

**Supplemental table 2. Classification of nutritional status based on Prognostic Nutritional Index**

|  | Prognostic Nutritional Index (PNI) | | |
| --- | --- | --- | --- |
|  | PNI < 40 | 40 ≤ PNI < 45 | PNI ≥ 45 |
| Clinical categories | Poor | Normal | Good |

PNI was calculated as 10 × serum albumin (g/dL) + 0.005 × total lymphocyte count (/mm³).
