# Supplementary material for: Prognostic significance of nutritional status for neurological and functional recovery after cervical spinal cord injury
Source: PLoS One. 2026 Jul 7;21(7):e0353302. doi: 10.1371/journal.pone.0353302 (PMC13340789; doi:10.1371/journal.pone.0353302)
Supplement: S3 Table — (DOCX) [file pone.0353302.s004.docx]

**Supplemental table 3. Classification of nutritional status based on Geriatric Nutritional Risk Index**

|  | Geriatric Nutritional Risk Index (GNRI) | | | |
| --- | --- | --- | --- | --- |
|  | GNRI < 82 | 82 ≤ GNRI < 92 | 92 ≤ GNRI < 98 | GNRI ≥ 98 |
| Clinical categories | Severe risk | Moderate risk | Low risk | No risk |

GNRI was calculated as (1.489 × serum albumin [g/L]) + (41.7 × present body weight / ideal body weight).
