# Supplementary material for: Prognostic significance of nutritional status for neurological and functional recovery after cervical spinal cord injury
Source: PLoS One. 2026 Jul 7;21(7):e0353302. doi: 10.1371/journal.pone.0353302 (PMC13340789; doi:10.1371/journal.pone.0353302)
Supplement: S4 Table — (DOCX) [file pone.0353302.s005.docx]

**Supplemental table 4. Controlling Nutritional Status (CONUT) score**

| Prognostic factor | Score |
| --- | --- |
| Serum albumin (g/dL) |  |
| ≥ 3.5 | 0 |
| 3.0 – 3.49 | 2 |
| 2.5 – 2.99 | 4 |
| < 2.5 | 6 |
| Total lymphocyte count (/mm³) |  |
| ≥ 1600 | 0 |
| 1200 – 1599 | 1 |
| 800 – 1199 | 2 |
| < 800 | 3 |
| Total cholesterol (mg/dL) |  |
| ≥ 180 | 0 |
| 140 – 179 | 1 |
| 100 – 139 | 2 |
| < 100 | 3 |

Each parameter was scored according to predefined cut-off values, and the sum of the three scores was used as the Controlling Nutritional Status (CONUT) score. Patients were categorized according to the CONUT score as follows: normal nutritional status (CONUT ≤ 1), mild malnutrition (2 ≤ CONUT < 5), moderate malnutrition (5 ≤ CONUT < 9), and severe malnutrition (CONUT ≥ 9).
