# Supplementary material for: Prognostic significance of nutritional status for neurological and functional recovery after cervical spinal cord injury
Source: PLoS One. 2026 Jul 7;21(7):e0353302. doi: 10.1371/journal.pone.0353302 (PMC13340789; doi:10.1371/journal.pone.0353302)
Supplement: S5 Table — (DOCX) [file pone.0353302.s006.docx]

**Supplemental table 5. Classification of nutritional status based on Controlling Nutritional Status**

|  | Controlling Nutritional Status (CONUT) | | | |
| --- | --- | --- | --- | --- |
|  | CONUT ≤ 1 | 2 ≤ CONUT < 5 | 5 ≤ CONUT < 9 | CONUT ≥ 9 |
| Clinical categories | Normal nutritional status | Mild malnutrition | Moderate malnutrition | Severe malnutrition |
