# Supplementary material for: Prognostic significance of nutritional status for neurological and functional recovery after cervical spinal cord injury
Source: PLoS One. 2026 Jul 7;21(7):e0353302. doi: 10.1371/journal.pone.0353302 (PMC13340789; doi:10.1371/journal.pone.0353302)
Supplement: S6 Table — (DOCX) [file pone.0353302.s007.docx]

**Supplemental table 6. Changes in ASIA Impairment Scale at 4 weeks and 6 months after SCI**

|  | | ASIA Impairment Scale 6 months after SCI | | | |
| --- | --- | --- | --- | --- | --- |
|  |  | A | B | C | D |
| ASIA Impairment Scale  4 weeks after SCI | A | 21 (84%) | 3 (12%) | 1 (4%) | 0 (0%) |
|  | B | 1 (6.67%) | 9 (60%) | 5 (33.33%) | 0 (0%) |
|  | C | 0 (0%) | 1 (4.76%) | 8 (38.1%) | 12 (57.14%) |
|  | D | 0 (0%) | 0 (0%) | 0 (0%) | 30 (100%) |

ASIA: American Spinal Injury Association; SCI: Spinal Cord Injury

Variables are given as the number with the percentage in parenthesis.
