# Supplementary material for: Prognostic significance of nutritional status for neurological and functional recovery after cervical spinal cord injury
Source: PLoS One. 2026 Jul 7;21(7):e0353302. doi: 10.1371/journal.pone.0353302 (PMC13340789; doi:10.1371/journal.pone.0353302)
Supplement: S7 Table — (DOCX) [file pone.0353302.s008.docx]

**Supplemental table 7. Changes in Prognostic Nutritional Index categories at 4 weeks and 6 months after SCI**

|  | | PNI categories 6 months after SCI | | |
| --- | --- | --- | --- | --- |
|  |  | Good | Normal | Poor |
| PNI categories  4 weeks after SCI | Good | 20 (90.91%) | 2 (9.09%) | 0 (0%) |
|  | Normal | 20 (71.43%) | 5 (17.86%) | 3 (10.71%) |
|  | Poor | 12 (29.27%) | 14 (34.15%) | 15 (36.59%) |

PNI: Prognostic Nutritional Index; SCI: Spinal Cord Injury

Variables are given as the number with the percentage in parenthesis. Supplemental table
