# Supplementary material for: Prognostic significance of nutritional status for neurological and functional recovery after cervical spinal cord injury
Source: PLoS One. 2026 Jul 7;21(7):e0353302. doi: 10.1371/journal.pone.0353302 (PMC13340789; doi:10.1371/journal.pone.0353302)
Supplement: S8 Table — (DOCX) [file pone.0353302.s009.docx]

**Supplemental table 8. Changes in Geriatric Nutritional Risk Index categories at 4 weeks and 6 months after SCI**

|  | | GNRI categories 6 months after SCI | | | |
| --- | --- | --- | --- | --- | --- |
|  |  | No risk | Low risk | Moderate risk | Severe risk |
| GNRI categories 4 weeks after SCI | No risk | 14 (82.35%) | 2 (11.76%) | 1 (5.88%) | 0 (0%) |
|  | Low risk | 11 (55%) | 7 (35%) | 2 (10%) | 0 (0%) |
|  | Moderate risk | 6 (16.22%) | 16 (43.24%) | 13 (35.14%) | 2 (5.41%) |
|  | Severe risk | 0 (%) | 7 (41.18%) | 8 (47.06%) | 2 (11.76%) |

GNRI: Geriatric Nutritional Risk Index; SCI: Spinal Cord Injury

Variables are given as the number with the percentage in parenthesis.
