# Supplementary material for: Prognostic significance of nutritional status for neurological and functional recovery after cervical spinal cord injury
Source: PLoS One. 2026 Jul 7;21(7):e0353302. doi: 10.1371/journal.pone.0353302 (PMC13340789; doi:10.1371/journal.pone.0353302)
Supplement: S9 Table — (DOCX) [file pone.0353302.s010.docx]

**Supplemental table 9. Changes in Controlling Nutritional Status categories at 4 weeks and 6 months after SCI**

|  | | CONUT categories 6 months after SCI | | | |
| --- | --- | --- | --- | --- | --- |
|  |  | Normal | Mild malnutrition | Moderate malnutrition | Severe malnutrition |
| CONUT categories 4 weeks after SCI | Normal | 18 (75%) | 5 (20.83%) | 1 (4.17%) | 0 (0%) |
|  | Mild malnutrition | 20 (60.61%) | 12 (36.36%) | 1 (3.03%) | 0 (0%) |
|  | Moderate malnutrition | 7 (23.33%) | 14 (46.67%) | 9 (30%) | 0 (0%) |
|  | Severe malnutrition | 1 (25%) | 1 (25%) | 2 (50%) | 0 (0%) |

CONUT: Controlling Nutritional Status; SCI: Spinal Cord Injury

Variables are given as the number with the percentage in parenthesis.
