# Supplementary material for: Prognostic significance of nutritional status for neurological and functional recovery after cervical spinal cord injury
Source: PLoS One. 2026 Jul 7;21(7):e0353302. doi: 10.1371/journal.pone.0353302 (PMC13340789; doi:10.1371/journal.pone.0353302)
Supplement: Attachment — Submitted filename: Response to Reviewers.docx [file pone.0353302.s013.docx]

To the editor and reviewers:

We appreciate the high quality and constructive review given to our manuscript. We have made every effort to carefully address all comments and suggestions in a point-by-point manner, as we believe they have helped to improve the clarity and scientific quality of our work. The reviewers’ comments are shown in bold face and our responses are typed below. The changes made in the revised manuscript (*italic face*) are indicated in underlined text. We would be pleased to provide further clarification should any points require additional explanation.

**Reviewer: 1**

**Reviewer #1: Manuscript Title: Prognostic Significance of Nutritional Status for Neurological and Functional Recovery after Cervical Spinal Cord Injury**

**Strengths**

**This study addresses a clinically relevant question by examining the association between early nutritional status and recovery after cervical SCI. The use of validated nutritional indices alongside standardized neurological and functional outcomes strengthens the analysis, and the focus on the early post-injury period enhances its clinical relevance. The identification of PNI as an independent predictor of recovery is particularly noteworthy and supports the potential role of nutritional assessment in early prognostic evaluation.**

We are sincerely grateful for your positive and encouraging comments. We are pleased that you recognized the clinical importance of evaluating nutritional status in the early phase after cervical spinal cord injury (SCI). As you noted, the early post-injury period is a critical time for recovery, and we believe that nutritional management is an important factor during this stage. We hope that our findings will highlight the role of early nutritional assessment and that this study will encourage clinicians to include nutritional optimization as an essential part of comprehensive care for SCI patients. We believe that integrating nutritional management into standard clinical practice can help improve patient outcomes.

**Weaknesses and Recommendations**

**Several aspects of the study would benefit from further clarification and refinement. There is some inconsistency in the terminology used to describe neurological outcomes (e.g., ASIA motor score, AIS, ISNCSCI), and clearer specification of the measures applied would enhance interpretability. Additional methodological detail would also strengthen the manuscript, particularly regarding the clinical context at 4 weeks (e.g., rehabilitation setting, standard nutritional support) and the definition of certain exclusion criteria such as “systemic deterioration.” The presentation of results could be streamlined to improve clarity and flow, with more consistent reporting of ranges and outcomes. While the inclusion of multiple nutritional indices is a strength, the use of BMI would benefit from acknowledgment of its known limitations in the SCI population, where it may not accurately reflect body composition or cardiometabolic risk. Finally, minor refinements in the conclusions and table organization could further improve overall readability.**

Thank you for these important suggestions. We agree that the terminology and certain clinical details needed more clarity and consistency. We have carefully revised the manuscript based on your feedback, and we believe these changes have made our study much easier to understand. First, we have standardized the terminology for neurological outcomes throughout the text to ensure consistency. We have also provided more detailed information in the Methods section regarding the rehabilitation setting, the standard nutritional support provided to our patients, and a clearer definition of the exclusion criterion systemic deterioration. Regarding BMI, we have added a section in the Discussion to acknowledge its limitations in the SCI population. Specifically, we noted that BMI may not accurately reflect actual body composition or cardiometabolic risk in these patients due to changes like muscle atrophy. Finally, we have refined the Results section and simplified the Conclusions to focus on the main findings, moving some of the interpretative text to the Discussion. The tables have also been reorganized for better readability.

We are very grateful for your insightful guidance. We believe that these revisions have significantly improved the scientific quality of our manuscript, and we hope it is now suitable for publication in *PLOS ONE*.

**Specific Comments:**

**Abstract:**

**It is mentioned that: “Neurological function was assessed using the American Spinal Injury Association (ASIA) motor score”….were only the motor scores used?? Or you meant the ASIA Impairment Scale (AIS)??**

We apologize for the confusion caused by our terminology. To clarify, the primary neurological outcome used in our analysis was the total motor score as defined by the International Standards for Neurological Classification of Spinal Cord Injury (ISNCSCI). This score represents the sum of the motor scores for both the upper and lower extremities. While the ASIA Impairment Scale (AIS) was used to describe the severity of the injury and the baseline characteristics of our patients, the recovery rates and the multivariable analysis were based on the ISNCSCI total motor score.

Recognizing that the term “ASIA motor score” was imprecise and potentially misleading, we have revised the entire manuscript (including the Abstract, Methods, Results, and Tables) to consistently use the term “ISNCSCI total motor score.” We believe this change clarifies that we focused on the quantitative motor recovery of the patients.

**Methods:**

**Could you please explain more, what do you mean by: “…..and 14 due to systemic deterioration.”**

We appreciate the opportunity to clarify the term "systemic deterioration." This exclusion criterion refers to patients who developed severe medical complications necessitating transfer to other specialized facilities or departments for acute intensive management, which prevented the evaluation of their 6-month outcomes. Specifically, these 14 cases included exacerbations of pneumonia or urinary tract infections (n=12), adhesive ileus (n=1), and septic shock following a surgical site infection (n=1). To improve clarity, we have added a definition of these complications to the Methods section .

*Page 8, line 68 in the Materials and Methods section*

*In this study, systemic deterioration was defined as the occurrence of severe medical complications that necessitated transfer to other facilities for acute intensive management, such as severe respiratory or urinary tract infections, acute abdominal conditions, or sepsis.*

**Line 78-79: “Neurological status was assessed using the American Spinal Injury Association 79 (ASIA) motor score..” do you mean ASIA impairment scale (AIS)? Or the international standards for neurological classification of spinal cord injury (ISNCSCI).**

We apologize for the lack of precision in our terminology. As the reviewer correctly pointed out, we used the total motor score defined by the International Standards for Neurological Classification of Spinal Cord Injury (ISNCSCI) as our primary neurological outcome. While the ASIA Impairment Scale (AIS) was used to classify the severity of the injury at baseline, the quantitative analysis of recovery and the multivariable models were based on the ISNCSCI total motor score (the sum of the upper and lower extremity motor scores).

In accordance with your suggestion, we have updated the terminology throughout the manuscript, including the Abstract, Methods, Results, and Tables, to consistently use "ISNCSCI total motor score." in the Methods section has been revised as follows.

*Page 9, line 101 in the Materials and Methods section*

*Neurological status was assessed using the International Standards for Neurological Classification of Spinal Cord Injury (ISNCSCI) total motor score, and functional independence was evaluated using the Spinal Cord Independence Measure (SCIM).*

**Line 82-83: I suggest using this language for AIS C definition “less than half of key muscle functions below the single NLI have a muscle grade ≥ 3.”**

We appreciate this constructive suggestion. We have revised the definition of AIS C in the Methods section (Line 82–83) to align with the standard terminology of the International Standards for Neurological Classification of Spinal Cord Injury (ISNCSCI), as you suggested.

*Page 9, line 103 in the Materials and Methods section*

*The severity of SCI was classified using the American Spinal Injury Association Impairment Scale (AIS), where AIS A indicates complete injury, AIS B sensory incomplete injury, AIS C motor incomplete injury with less than half of key muscle functions below the single neurological level of injury (NLI) have a Manual Muscle Test (MMT) grade ≥ 3, and AIS D motor incomplete injury with at least half of the key muscles having an MMT grade ≥ 3.*

**Line 98: “the CONUT score, an indicator of nutritional and immunological status,”….in the intro it is mentioned that CONUT score captures both protein and energy deficiency. Please clarify.**

We appreciate the reviewer's careful reading. The CONUT score is designed to assess the degree of malnutrition by evaluating protein reserves (serum albumin), energy balance (total cholesterol), and immune defense (total lymphocyte count). Therefore, it serves as a comprehensive indicator of both nutritional (protein-energy) and immunological status. To ensure consistency, we have revised the description in both the Introduction and Methods sections to clarify that the CONUT score captures protein-energy deficiency as well as immunological status.

*Page 7, line 50 in the Introduction section*

*CONUT, which incorporates serum albumin, lymphocyte count, and total cholesterol, allows rapid nutritional screening without requiring anthropometric measurements and captures nutritional (protein-energy) as well as immunological status.*

*Page 10, line 120 in the Materials and Methods section*

*The CONUT score, an indicator of nutritional (protein-energy) and immunological status, was calculated based on serum albumin concentration, total lymphocyte count, and total cholesterol level.*

**Line 106: “ASIA motor score” please change to ISNCSCI motor scores” (and modify everywhere where it is applicable)…..Consider using also the terminology of “Total motor scores (TMS) or Upper extremity motor scores (UEMS)”, depending on which motor scores you are using.;**

We are grateful for this specific guidance regarding the nomenclature. In accordance with your recommendation, we have replaced all instances of “ASIA motor score” with “ISNCSCI total motor score” throughout the entire manuscript, including the Abstract, Methods, Results, Discussion, Tables, and Figures. Since our analysis focused on overall neurological recovery, we have consistently used the total motor score (the sum of the upper and lower extremity motor scores) as the primary outcome measure to ensure clarity and scientific accuracy.

**Periods (.) are missing after “100” in line 111 and line 114.**

We appreciate the reviewer’s meticulous attention to detail. We have added the missing periods after "100" in the formulas for the recovery rates on lines 111 and 114 (in the revised manuscript, these are now on lines 144 and 148).

**Please elaborate as part of the methods, is the patients at 4 weeks were receiving inpatient acute rehab or outpatient. If as part of the regular treatment they receive any nutritional support?? Or what is the standard protocol regarding nutrition and you institution.**

We appreciate this important request for clarification regarding the clinical setting and the standard of care. Regarding the rehabilitation setting, all 91 patients included in this study were receiving comprehensive inpatient rehabilitation at the 4-week mark and remained hospitalized for at least 6 months. There were no outpatient participants. Rehabilitation was provided by a multidisciplinary team (physicians, physical therapists, occupational therapists, and nurses) and was tailored to each patient's injury level and severity (AIS grade) based on our institutional clinical pathways.

Regarding nutritional support, our institution follows a standardized protocol managed by a Nutrition Support Team (NST). Patients are screened monthly using the Malnutrition Universal Screening Tool (MUST). Those identified as being at risk (MUST score ≥ 2) undergo a formal malnutrition diagnosis based on the Global Leadership Initiative on Malnutrition (GLIM) criteria, which incorporates phenotypic factors (weight loss, low BMI, and reduced muscle mass measured by bioelectrical impedance analysis) and etiologic factors (reduced food intake/assimilation and inflammation due to acute injury).

Nutritional interventions were individualized based on these assessments and included the use of high-protein/high-calorie oral nutritional supplements or enteral nutrition as needed to meet the patient’s metabolic demands during the subacute phase. We have added these details to the Methods section.

*Page 8, line 72 in the Materials and Methods section*

*All participants received specialized inpatient rehabilitation at our spinal injury center. At the 4-week post-injury assessment, all patients were undergoing a comprehensive multidisciplinary rehabilitation program. The rehabilitation program followed a standardized institutional protocol, which is summarized in Supplemental Table 1. This program involved a systematic progression from acute stabilization and bedside therapy to comprehensive functional training, including mobility exercises and ADL. Throughout the inpatient stay, patients received daily physical and occupational therapy tailored to the neurological level and severity of their injury to ensure a consistent approach to recovery.*

*Page 8, line 80 in the Materials and Methods section*

*Nutritional status was managed according to a standardized institutional protocol overseen by a Nutrition Support Team (NST). All patients underwent regular nutritional screening using the Malnutrition Universal Screening Tool (MUST). For patients identified as being at risk (MUST score ≥ 2), a formal diagnosis of malnutrition and its severity was performed based on the Global Leadership Initiative on Malnutrition (GLIM) criteria. Phenotypic assessment included monitoring of unintentional weight loss, Body Mass Index (BMI), and skeletal muscle mass index (SMI) measured via bioelectrical impedance analysis. Etiologic assessment focused on reduced oral intake and the presence of systemic inflammation following acute trauma. Based on these evaluations, individualized nutritional support was provided, including protein-rich oral nutritional supplements or enteral nutrition, to address the hypermetabolic state associated with the subacute phase of SCI.*

**Results:**

**Line 148-150: with this statement I am assuming you used total motor scores, but it’s until now that I realize this. Please specify form the beginning and adjust accordingly.**

We sincerely apologize for the lack of clarity in our initial manuscript regarding the specific neurological metrics used. We appreciate the reviewer pointing this out. As per your suggestion, we have systematically revised the entire manuscript to explicitly specify that the ISNCSCI total motor score (representing the sum of the motor scores for both upper and lower extremities) was used as our primary outcome measure. This terminology is now consistently applied from the beginning of the Abstract through the Methods, Results, and Tables to ensure that the reader can clearly follow our analysis of neurological recovery.

**If you used SCIM III version, please specify from the very beginning.**

We thank the reviewer for this important point. We confirm that the Spinal Cord Independence Measure version III (SCIM III) was used for all functional assessments in this study. In accordance with your suggestion, we have specified this from the beginning of the manuscript, including the Abstract and the Methods section, and ensured that the terminology is consistently used throughout the text.

**Line 153-155: Please check that the ranges in parentheses are correct: “The mean change in 154 SCIM score was 21.6 ± 18.22 (range, −2 – 80), with a mean SCIM recovery rate of 27.96 ± 155 24.84% (range, −2.38% – 95.74%) (Table 1)”**

We appreciate the reviewer's careful check of our data. We have re-verified the raw data and the calculations, and we confirm that the reported values and ranges are correct. The negative values (range: -2 for SCIM III change and -2.38% for SCIM III recovery rate) reflect a few specific cases where the functional score showed a slight decrease between the 4-week and 6-month assessments due to medical complications or fluctuations in the patient's general condition. We believe that including these cases is necessary for an accurate and transparent representation of the clinical outcomes in our cohort.

**Line 161-168: I suggest adding all the percentages.**

Thank you for this helpful suggestion. We have added the corresponding percentages to all the patient counts in the Results section (specifically lines 161–168 and throughout the paragraph) to improve clarity and facilitate comparison between different nutritional indices.

**The paragraph on “Nutritional status at 4 weeks and 6 months after SCI”….is a bit confusing, try to present the results in order for pre and post by each outcome measure, other wise the reader has to move up and down to see the pre and post values.**

We apologize for the confusion caused by the structure of the Results section. We understand that presenting all the 4-week data followed by all the 6-month data made it difficult to compare the progression of each index. Following your advice, we have reorganized this paragraph to present the results by each nutritional index, showing the 4-week and 6-month values consecutively. We believe this structure much more clearly illustrates the changes in nutritional status over time.

*Page 15, line 220 in the Results section*

*Changes in nutritional status between 4 weeks and 6 months after SCI*

*Detailed longitudinal shifts in nutritional status for each index are summarized in Supplemental Tables 6–8. Overall, nutritional status improved in a substantial proportion of patients across all three indices, while deterioration was observed in only a minority of cases.*

*PNI: Among patients classified as having good nutritional status at 4 weeks, 90.91% (20/22) remained in the same category at 6 months. Of those classified as normal at 4 weeks, 71.43% (20/28) improved to the good category at 6 months, whereas only 10.71% (3/28) deteriorated to the poor category. Notably, 63.41% (26/41) of patients in the poor group at 4 weeks showed improvement at 6 months, with 29.27% (n=12) reaching the good category and 34.15% (n=14) moving to normal.*

*GNRI: Regarding nutritional risk, 82.35% (14/17) of the no risk group at 4 weeks remained stable at 6 months. Among patients at moderate risk at 4 weeks, 59.46% (22/37) improved to either the no-risk or low-risk category at 6 months, while only 5.4% (2/37) progressed to severe risk. Additionally, 88.24% (15/17) of those initially at severe risk improved to the low-risk or moderate-risk category at 6 months.*

*CONUT score: Similar positive trends were observed for the CONUT score. Of the patients with mild malnutrition at 4 weeks, 60.61% (20/33) improved to the normal category at 6 months. Furthermore, 70% (21/30) of patients with moderate malnutrition at 4 weeks improved to either normal or mild malnutrition at 6 months. Importantly, no patients were classified as having severe malnutrition at 6 months.*

**The “Multivariable linear regression analyses for neurological and functional recovery” paragraph is a bit hard to follow; please consider re-ordering the information and avoiding repetition.**

We appreciate this feedback. We have reorganized the Results section regarding the multivariable linear regression analyses to improve clarity and flow. Specifically, we first defined the common covariates for both models to avoid repetition and then presented the results for neurological and functional recovery separately. We also refined the sentence structures to highlight the key independent predictors more effectively. We believe these changes make the findings much easier to follow.

*Page 17, line 290 in the Results section*

*Multivariable linear regression analyses for neurological and functional recovery*

*Multivariable linear regression analyses were conducted to identify independent predictors of recovery, with the models for neurological recovery (ISNCSCI total motor score improvement rate) and functional recovery (SCIM III recovery rate) summarized in Tables 4 and 5. Both models included age, sex, and 4-week baseline values for PNI, ISNCSCI total motor score, and SCIM III score as covariates.*

*For neurological recovery, the model explained 47.54% of the variance (R^2^ = 0.4754). After adjusting for potential confounders, PNI at 4 weeks remained independently associated with the total motor score improvement rate (β = 0.857 per 1-point increase, 95% CI 0.011–1.703; p = 0.0471). Additionally, a higher baseline motor score was strongly associated with greater improvement (β = 0.724 per 1-point increase, 95% CI 0.488–0.961; p < 0.0001). In this model, age, sex, and baseline SCIM III score did not show independent associations.*

*For functional recovery, the model showed that PNI at 4 weeks was not an independent predictor of SCIM III improvement (β = -0.02, 95% CI -0.676 to 0.634; p = 0.9503). Instead, older age was significantly associated with a lower recovery rate (β = -0.275 per year, 95% CI -0.531 to -0.018; p = 0.0362), while a higher baseline motor score was independently associated with greater functional gains (β = 0.767 per 1-point increase, 95% CI 0.584–0.950; p < 0.0001). Sex and baseline SCIM III score showed no significant independent associations with the outcome.*

**Conclusions:**

**This sentence in conclusions: “Although nutritional status was not an independent predictor of functional recovery assessed by SCI after multivariable adjustment, its significant associations with neurological improvement suggest that nutritional status may influence functional recovery indirectly through its effects on neurological recovery.” Belongs to the discussion, I don’t think this is a conclusion**

We agree with the reviewer’s assessment. The sentence regarding the potential indirect influence of nutritional status on functional recovery was indeed interpretative and is more appropriate for the Discussion section. We have removed this sentence from the Conclusions and revised the section to focus on the primary findings and their clinical implications. We have ensured that the discussion of indirect effects is appropriately addressed in the Discussion section.

*Page 26, line 458 in the Conclusions section*

*Conclusions*

*In patients with cervical SCI, nutritional status assessed at 4 weeks after injury was significantly associated with subsequent recovery outcomes. Among the indices evaluated, the PNI emerged as the most informative marker, showing a consistent and independent association with neurological recovery as measured by the ISNCSCI total motor score improvement rate. These findings highlight the importance of early nutritional status in the recovery process and suggest that nutritional assessment and optimization should be integrated into the comprehensive care of patients after cervical SCI to maximize their recovery potential.*

**Tables:**

**Could table 4 and 5 be condensed in one table?**

We agree with the reviewer that Tables 4 and 5 were redundant. We have condensed them into a single table (now Table 4) that presents the multivariable linear regression results for both ISNCSCI total motor score and SCIM III score improvement rates side-by-side. This reorganization allows for an easier comparison of the different predictors for neurological versus functional recovery.

We are very grateful for the time and effort you put into reviewing our study. Your feedback was essential in helping us clarify our methodology and standardize the terminology throughout the manuscript. We feel that these revisions have made the paper much stronger and more accessible to the readers.

We have carefully integrated all your suggestions and hope that the revised version meets your approval for publication in *PLOS ONE*. Thank you again for your valuable guidance in improving our work.

_________________________________________________________________________________

**Reviewer #2: Congratulations.**

**A very interesting cohort study correlating nutritional status with cervical spinal cord injury.**

Thank you for your positive feedback. We are pleased that you found our study on the relationship between nutritional status and cervical spinal cord injury interesting.

**I have a question: among the patients included in this clinical research, were there any subject with a metabolic or endocrine disorder?**

Thank you for this insightful question. In response to your comment, we have performed a detailed review of the patients' medical records regarding metabolic and endocrine comorbidities.

Among the 91 included patients, 17 patients (18.68%) had diabetes mellitus, all of whom were clinically stable during the study period. No patients were identified with other specific endocrine disorders, such as thyroid dysfunction, or chronic hepatic diseases that significantly impact systemic metabolism.

Regarding renal function, which may influence both nutritional markers, we assessed the baseline estimated glomerular filtration rate (eGFR). The distribution was as follows: 73 patients had an eGFR ≥ 60 mL/min/1.73 m², 18 patients had an eGFR of 30–60 mL/min/1.73 m², and no patients had an eGFR < 30 mL/min/1.73 m².

We have added these clinical characteristics to the Results section and Table 1 to provide a more comprehensive description of our cohort.

*Page 13, line 179 in the Results section*

*Regarding comorbidities, 17 patients (18.68%) had diabetes mellitus; however, all were clinically stable and received appropriate medical management throughout their hospitalization. No other specific metabolic or endocrine disorders, such as thyroid dysfunction or chronic hepatic disease, were identified in our cohort. Baseline renal function assessment showed that 73 patients (80.22%) had an estimated glomerular filtration rate (eGFR) ≥ 60 mL/min/1.73 m^2^, while 18 patients (19.78%) had an eGFR between 30 and 60 mL/min/1.73 m^2^; no patients had an eGFR < 30 mL/min/1.73 m^2^.*

**It would also be interesting to continue this type of study and include nutritional management during the follow-up period as a controlled variable.**

We agree that incorporating specific nutritional management protocols as a controlled variable is a critical next step for this field of research. In our current retrospective study, while all patients received standardized nutritional support according to our institutional protocol, we did not analyze the specific caloric or protein intake as independent variables. As you suggested, future prospective studies should incorporate detailed nutritional interventions as controlled variables to further elucidate the direct impact of specific management strategies on neurological and functional recovery. We have added this perspective to the Discussion section.

*Page 24, line 444 in the Discussion section*

*Fourth, we did not collect detailed data on the specific nutritional interventions, such as precise caloric and protein intake, for each patient throughout the follow-up period. Consequently, we were unable to include nutritional management as a controlled variable in our analysis to evaluate its direct impact. Future prospective studies incorporating these management details are needed to clarify the relationship between specific nutritional support and recovery.*

Thank you for your positive comments and for highlighting the importance of comorbidities. Following your suggestion, we have included more detailed information regarding the metabolic and endocrine profiles of our patients, which we believe helps to better describe the study population.

We have carefully considered all your feedback and hope that the additional data and clarifications make the manuscript more comprehensive. Thank you again for your time and for helping us improve our work.

_________________________________________________________________________________

**Reviewer #3: In this retrospective study, the authors investigated the prognostic value of early nutritional indices - the Prognostic Nutritional Index (PNI), Geriatric Nutritional Risk Index (GNRI), and Controlling Nutritional Status (CONUT) score - in predicting recovery outcomes for patients with traumatic cervical spinal cord injury (SCI). The study highlights a modifiable factor in rehabilitation of patients with cervical spinal cord injury. The authors have utilized appropriate multivariate liner regression tools to control for confounders. The manuscript is well-structured, and the results have been compiled appropriately. However, the following major and minor flaws remain that need to be addressed:**

We thank the reviewer for the positive assessment of our study and for recognizing the clinical importance of identifying modifiable factors in SCI rehabilitation. Your detailed feedback on the major and minor points was very helpful. We have made every effort to address each concern by providing additional data and expanding the Discussion section. We believe that these revisions have added significant depth and clarity to the manuscript.

**1. Methods: Please explain how 4 weeks post injury time interval was selected as “early nutritional status” in this study vs. on admission? Is this a standard timeline for determining early nutritional status in spinal cord injury patients? Discuss the rationale for selecting this cut off in the methods.**

We thank the reviewer for this critical point regarding the timing of the nutritional assessment. We selected the 4-week mark as the baseline for early nutritional status rather than the time of admission for the following reasons.

First, in the immediate post-injury period (admission to approximately 2 weeks), biochemical markers such as serum albumin and total lymphocyte count are significantly affected by the systemic inflammatory response and surgical stress. During this acute phase, albumin functions as a negative acute-phase reactant, reflecting the severity of physiological stress rather than the patient's underlying nutritional status.

Second, by 4 weeks post-injury, patients have typically completed acute medical and surgical stabilization and have transitioned into a phase of consistent, intensive rehabilitation. This interval serves as a more reliable clinical baseline for prognostic evaluation, as it reflects the patient's nutritional standing at the start of the long-term recovery process. Previous studies have also indicated that neurological assessments performed around 1 month post-injury are more predictive of long-term outcomes than those performed in the first few weeks.

We have added this rationale and the supporting literature to the Methods section.

*Page 10, line 127 in the Materials and Methods section*

*Nutritional status was evaluated at 4 weeks and 6 months after injury. The 4-week time point was selected as the baseline for early nutritional status because biochemical markers such as serum albumin are strongly influenced by the acute inflammatory response and surgical stress during the hyper-acute phase after spinal cord injury.[19, 20] Consequently, assessments performed immediately after injury may primarily reflect physiological stress responses rather than the patient’s underlying nutritional condition. Previous studies have demonstrated that the prognostic value of serum albumin measurements increases progressively over the first month after injury, with 4-week assessments showing stronger associations with long-term neurological outcomes than earlier measurements.[21] In addition, by 4 weeks post-injury, most patients have completed acute medical stabilization and entered the intensive rehabilitation phase, providing a more stable baseline for prognostic evaluation.*

**2. What was the mean length of stay for 206 eligible patients?**

**3. The study started with 206 eligible patients but excluded 94 due to early discharge/transfer. The authors must address whether the "early discharge" group differed significantly from the "included" group. If the healthier patients were discharged early, the remaining cohort might be skewed toward more severe cases, biasing the results.**

We are truly grateful for these thoughtful and critical questions regarding our cohort selection and the potential for bias. As these two points are related, we have merged our response to provide a more comprehensive explanation.

Regarding the length of stay (LOS), the mean LOS for the 91 patients included in the final analysis was 322.95 ± 90.2 days (range, 182–591 days). While we do not have complete day-by-day LOS data for all the initially screened 206 patients, the 94 excluded individuals were those who were discharged or transferred to local facilities before the 6-month follow-up assessment could be completed.

As the reviewer correctly pointed out, this exclusion criteria likely introduced a selection bias. The early discharge group primarily included patients with milder injuries who achieved faster functional stabilization. Consequently, our final cohort of 91 patients represents a more severe subpopulation of cervical SCI requiring intensive, long-term inpatient rehabilitation.

While this limits the generalizability of our findings to milder SCI cases, we believe that focusing on this long-term population is clinically significant. These patients are at the greatest risk of muscle atrophy and malnutrition, making prognostic assessment particularly important in this group. We have added this LOS data to Table 1 and included a detailed discussion of this selection bias in the Limitations section.

*Page 24, line 433 in the Discussion section*

*Second, a significant selection bias may exist because we excluded 94 patients who were discharged or transferred before the 6-month follow-up. These excluded patients often had milder injuries and better initial recovery, allowing for earlier discharge. As a result, our final cohort of 91 patients, who had an average length of stay of over 300 days, represents a more severe subpopulation of cervical SCI requiring intensive, long-term inpatient care. While this may limit the generalizability of our results to patients with milder SCI, the findings remain highly relevant for the management of patients with severe injuries who are at the highest risk for malnutrition and long-term disability.*

**4. Serum albumin is a negative acute phase reactant in critically ill patients and hence may influence PNI. A low PNI may simply be a proxy for a high "complication burden" (pneumonia, UTI) rather than nutritional intake itself. Please discuss under limitations of using PNI as a nutritional index.**

We fully agree with the reviewer's insightful observation. As you pointed out, serum albumin is a negative acute-phase reactant, and the total lymphocyte count is also sensitive to systemic stress. Therefore, a low PNI at 4 weeks may not only reflect a deficit in nutritional intake but also serve as a proxy for the patient's "complication burden," such as pneumonia, urinary tract infections (UTIs), or pressure ulcers, which are common after cervical SCI.

However, we believe this is precisely why PNI is a valuable prognostic marker; it captures the complex interplay between malnutrition and the inflammatory state caused by secondary complications. While PNI may not be a "pure" measure of nutritional intake, it effectively represents the patient's overall biological resilience and capacity for neural repair. We have added a new paragraph to the Discussion section to address this point and emphasized the need for more objective nutritional assessment tools that are less influenced by the acute-phase response.

*Page 21, line 359 in the Discussion section*

*Challenges of PNI as a Nutritional Assessment Tool*

*The prognostic value of PNI likely stems from its ability to reflect both protein-energy status and the systemic inflammatory burden. It is well-established that serum albumin is a negative acute-phase reactant, and its levels decrease in response to systemic inflammation regardless of nutritional intake.[32, 33] Patients with cervical SCI are particularly susceptible to secondary complications, such as pneumonia, urinary tract infections (UTIs), and pressure ulcers, all of which trigger a persistent inflammatory state.[34, 35] Consequently, a low PNI in the subacute phase may function as a surrogate marker for a high complication burden, which hinders neurological recovery. While PNI remains a useful clinical tool, our findings highlight the need for more objective and comprehensive nutritional assessment methods, such as bioelectrical impedance analysis or muscle ultrasound, that can distinguish between primary malnutrition and inflammation-induced metabolic changes.*

**5. The study did not mention the type of nutritional support received (TPN, enteral, or oral) during the first few weeks post injury. Differences in feeds and mode of providing nutrition could be a significant confounding variable that influences both the PNI and the recovery rate. Please address this under Discussion.**

We thank the reviewer for this important point. We agree that the way nutrition is provided (oral, enteral, or parenteral) and the specific amount of calories and protein are important factors that can influence both PNI levels and recovery rates.

In our study, all patients were managed by a Nutrition Support Team (NST) following a standard hospital protocol. This protocol prioritizes giving nutrition by mouth (oral) or through a tube (enteral) to support gut health and control inflammation. Intravenous (parenteral) nutrition was very rarely used in our cohort. However, we admit that we do not have detailed, individual data on the exact feeding methods and daily intake for each patient during the first few weeks.

As you suggested, we have added a discussion about this in the section "Nutritional Support for Neural Repair and Muscle Preservation." We have also clearly stated in the Limitations that the lack of this detailed information is a possible confounding factor in our study.

*Page 22, line 382 in the Discussion section*

*In addition to the total intake, the specific route of nutritional support, such as oral, enteral, or parenteral delivery, can also influence the systemic inflammatory environment. At our institution, patients were managed according to a standardized protocol by a Nutrition Support Team, which prioritizes early enteral or oral nutrition to maintain gut integrity and modulate inflammation. However, since specific data on feeding routes and daily dosages for each patient were not collected in this retrospective study, we could not analyze these factors as potential confounding variables. Given that different nutritional strategies can affect both the biochemical markers in the PNI and the recovery rates, this remains an important area for future prospective investigation.*

*Page 24, line 449 in the Discussion section*

*In addition, we did not analyze individual data regarding the specific mode of nutritional delivery (oral, enteral, or total parenteral nutrition) or the exact caloric and protein dosages provided during the initial post-injury weeks. Because the route and content of nutrition can significantly influence inflammatory markers and metabolic recovery, the lack of these detailed records remains a confounding factor in our analysis.*

**6. In the Results (Line 162), the PNI categories are listed as "good, normal, and poor," while the Methods (Line 90) use "normal, mild, and moderate to severe." Please check and make necessary corrections to ensure consistency throughout the manuscript including tables.**

We sincerely apologize for this inconsistency. The classification labels used in the Methods section were incorrect. We have corrected the Methods section to match the Results and Tables. Throughout the entire manuscript, including the Supplemental Tables, we now consistently use the following categories for PNI: "good" (PNI ≥ 45), "normal" (40 ≤ PNI < 45), and "poor" (PNI < 40) nutritional status.

*Page 10, line 113 in the Materials and Methods section*

*Patients were categorized based on the PNI as follows: good (PNI ≥ 45), normal (40 ≤ PNI < 45), and poor (PNI < 40) nutritional status (Supplemental Table 2).*

**7. Due to retrospective nature of the study, poor nutritional status as a cause for poor neurological recovery cannot be determined. Low PNI may represent an inflammatory state especially in patients with complications (pneumonia, pressure ulcers, etc.) post injury. The conclusions should explicitly suggest that PNI may be a useful prognostic marker for neurological and functional recovery.**

We thank the reviewer for this crucial comment. We agree that as a retrospective observational study, we cannot establish a causal relationship between nutritional status and neurological recovery. We acknowledge that a low PNI may indeed reflect a high complication burden and an inflammatory state rather than being the sole cause of poor recovery.

Following your advice, we have revised the Conclusions to explicitly state that the PNI is a useful prognostic marker for neurological recovery. We have also added a dedicated section in the Discussion titled "Challenges of PNI as a Nutritional Assessment Tool" and updated the Limitations section to clarify that a low PNI may serve as a marker of injury severity and complications. We believe these revisions provide a more accurate and balanced interpretation of our findings.

*Page 21, line 359 in the Discussion section*

*The prognostic value of PNI likely stems from its ability to reflect both protein-energy status and the systemic inflammatory burden. It is well-established that serum albumin is a negative acute-phase reactant, and its levels decrease in response to systemic inflammation regardless of nutritional intake.[32, 33] Patients with cervical SCI are particularly susceptible to secondary complications, such as pneumonia, UTIs, and pressure ulcers, all of which trigger a persistent inflammatory state.[34, 35] Consequently, a low PNI in the subacute phase may function as a surrogate marker for a high complication burden, which hinders neurological recovery. While PNI remains a useful clinical tool, our findings highlight the need for more objective and comprehensive nutritional assessment methods, such as bioelectrical impedance analysis or muscle ultrasound, that can distinguish between primary malnutrition and inflammation-induced metabolic changes.*

*Page 24, line 429 in the Discussion section*

*First, as an observational study, we cannot establish causality; a low PNI may be a marker of injury severity and complications rather than a direct cause of poor recovery. Specifically, a low PNI might represent a persistent inflammatory state associated with secondary complications such as pneumonia or pressure ulcers, which are common after cervical SCI.*

Thank you again for the rigorous and thoughtful review of our manuscript. Addressing your questions regarding our methodology and the clinical interpretation of the nutritional indices has helped us provide a much clearer rationale for our work and a more balanced presentation of the results.

We believe the manuscript is now much stronger thanks to your guidance, and we hope the revised version meets your approval for publication in *PLOS ONE*. Thank you for your time and for helping us improve the quality of this study.

_________________________________________________________________________________
