# Supplementary material for: Prognostic significance of nutritional status for neurological and functional recovery after cervical spinal cord injury
Source: PLoS One. 2026 Jul 7;21(7):e0353302. doi: 10.1371/journal.pone.0353302 (PMC13340789; doi:10.1371/journal.pone.0353302)
Supplement: Peer review history

# Peer review: Decision Letter 0

**DOI:** 10.1371/journal.pone.0353302.r001

**Authors:** Zubing Mei

16 Apr 2026

PONE-D-26-09186

Prognostic Significance of Nutritional Status for Neurological and Functional Recovery after Cervical Spinal Cord Injury

PLOS One

Dear Dr. Yokota,

Thank you for submitting your manuscript to PLOS ONE. After careful consideration, we feel that it has merit but does not fully meet PLOS ONE’s publication criteria as it currently stands. Therefore, we invite you to submit a revised version of the manuscript that addresses the points raised during the review process.

Please submit your revised manuscript by May 31 2026 11:59PM. If you will need more time than this to complete your revisions, please reply to this message or contact the journal office at plosone@plos.org. When you're ready to submit your revision, log on to https://www.editorialmanager.com/pone/ and select the 'Submissions Needing Revision' folder to locate your manuscript file.

Please include the following items when submitting your revised manuscript:

- A letter that responds to each point raised by the academic editor and reviewer(s). You should upload this letter as a separate file labeled 'Response to Reviewers'.
- A marked-up copy of your manuscript that highlights changes made to the original version. You should upload this as a separate file labeled 'Revised Manuscript with Track Changes'.
- An unmarked version of your revised paper without tracked changes. You should upload this as a separate file labeled 'Manuscript'.

If you would like to make changes to your financial disclosure, please include your updated statement in your cover letter. Guidelines for resubmitting your figure files are available below the reviewer comments at the end of this letter.

If applicable, we recommend that you deposit your laboratory protocols in protocols.io to enhance the reproducibility of your results. Protocols.io assigns your protocol its own identifier (DOI) so that it can be cited independently in the future. For instructions see: https://journals.plos.org/plosone/s/submission-guidelines#loc-laboratory-protocols. Additionally, PLOS ONE offers an option for publishing peer-reviewed Lab Protocol articles, which describe protocols hosted on protocols.io. Read more information on sharing protocols at https://plos.org/protocols?utm_medium=editorial-email&utm_source=authorletters&utm_campaign=protocols.

As the corresponding author, your ORCID iD is verified in the submission system and will appear in the published article. PLOS supports the use of ORCID, and we encourage all coauthors to register for an ORCID iD and use it as well. Please encourage your coauthors to verify their ORCID iD within the submission system before final acceptance, as unverified ORCID iDs will not appear in the published article. *Only*  the individual author can complete the verification step; PLOS staff *cannot*  verify ORCID iDs on behalf of authors.

We look forward to receiving your revised manuscript.

Kind regards,

Zubing Mei, MD,PH.D

Academic Editor

PLOS One

Journal requirements:

When submitting your revision, we need you to address these additional requirements.

1. Please ensure that your manuscript meets PLOS ONE's style requirements, including those for file naming. The PLOS ONE style templates can be found at

https://journals.plos.org/plosone/s/file?id=wjVg/PLOSOne_formatting_sample_main_body.pdf and

https://journals.plos.org/plosone/s/file?id=ba62/PLOSOne_formatting_sample_title_authors_affiliations.pdf

2. Your ethics statement should only appear in the Methods section of your manuscript. If your ethics statement is written in any section besides the Methods, please delete it from any other section.

If the reviewer comments include a recommendation to cite specific previously published works, please review and evaluate these publications to determine whether they are relevant and should be cited. There is no requirement to cite these works unless the editor has indicated otherwise.

[Note: HTML markup is below. Please do not edit.]

Reviewers' comments:

Reviewer's Responses to Questions

**Comments to the Author**

1. Is the manuscript technically sound, and do the data support the conclusions?

The manuscript must describe a technically sound piece of scientific research with data that supports the conclusions. Experiments must have been conducted rigorously, with appropriate controls, replication, and sample sizes. The conclusions must be drawn appropriately based on the data presented.

Reviewer #1: Yes

Reviewer #2: Yes

Reviewer #3: Yes

**********

2. Has the statistical analysis been performed appropriately and rigorously?

Reviewer #1: Yes

Reviewer #2: Yes

Reviewer #3: Yes

**********

3. Have the authors made all data underlying the findings in their manuscript fully available?

The PLOS Data policy requires authors to make all data underlying the findings described in their manuscript fully available without restriction, with rare exception (please refer to the Data Availability Statement in the manuscript PDF file). The data should be provided as part of the manuscript or its supporting information, or deposited to a public repository. For example, in addition to summary statistics, the data points behind means, medians and variance measures should be available. If there are restrictions on publicly sharing data—e.g. participant privacy or use of data from a third party—those must be specified.

Reviewer #1: Yes

Reviewer #2: Yes

Reviewer #3: Yes

**********

4. Is the manuscript presented in an intelligible fashion and written in standard English?

PLOS ONE does not copyedit accepted manuscripts, so the language in submitted articles must be clear, correct, and unambiguous. Any typographical or grammatical errors should be corrected at revision, so please note any specific errors here.

Reviewer #1: Yes

Reviewer #2: Yes

Reviewer #3: Yes

**********

5. Review Comments to the Author

Please use the space provided to explain your answers to the questions above. You may also include additional comments for the author, including concerns about dual publication, research ethics, or publication ethics. (Please upload your review as an attachment if it exceeds 20,000 characters)

Reviewer #1: Manuscript Title: Prognostic Significance of Nutritional Status for Neurological and Functional Recovery after Cervical Spinal Cord Injury

Strengths

This study addresses a clinically relevant question by examining the association between early nutritional status and recovery after cervical SCI. The use of validated nutritional indices alongside standardized neurological and functional outcomes strengthens the analysis, and the focus on the early post-injury period enhances its clinical relevance. The identification of PNI as an independent predictor of recovery is particularly noteworthy and supports the potential role of nutritional assessment in early prognostic evaluation.

Weaknesses and Recommendations

Several aspects of the study would benefit from further clarification and refinement. There is some inconsistency in the terminology used to describe neurological outcomes (e.g., ASIA motor score, AIS, ISNCSCI), and clearer specification of the measures applied would enhance interpretability. Additional methodological detail would also strengthen the manuscript, particularly regarding the clinical context at 4 weeks (e.g., rehabilitation setting, standard nutritional support) and the definition of certain exclusion criteria such as “systemic deterioration.” The presentation of results could be streamlined to improve clarity and flow, with more consistent reporting of ranges and outcomes. While the inclusion of multiple nutritional indices is a strength, the use of BMI would benefit from acknowledgment of its known limitations in the SCI population, where it may not accurately reflect body composition or cardiometabolic risk. Finally, minor refinements in the conclusions and table organization could further improve overall readability.

Specific Comments:

Abstract:

It is mentioned that: “Neurological function was assessed using the American Spinal Injury Association (ASIA) motor score”….were only the motor scores used?? Or you meant the ASIA Impairment Scale (AIS)??

Methods:

Could you please explain more, what do you mean by: “…..and 14 due to systemic deterioration.”

Line 78-79: “Neurological status was assessed using the American Spinal Injury Association 79 (ASIA) motor score..” do you mean ASIA impairment scale (AIS)? Or the international standards for neurological classification of spinal cord injury (ISNCSCI).

Line 82-83: I suggest using this language for AIS C definition “less than half of key muscle functions below the single NLI have a muscle grade ≥ 3.”

Line 98: “the CONUT score, an indicator of nutritional and immunological status,”….in the intro it is mentioned that CONUT score captures both protein and energy deficiency. Please clarify.

Line 106: “ASIA motor score” please change to ISNCSCI motor scores” (and modify everywhere where it is applicable)…..Consider using also the terminology of “Total motor scores (TMS) or Upper extremity motor scores (UEMS)”, depending on which motor scores you are using.;

Periods (.) are missing after “100” in line 111 and line 114.

Please elaborate as part of the methods, is the patients at 4 weeks were receiving inpatient acute rehab or outpatient. If as part of the regular treatment they receive any nutritional support?? Or what is the standard protocol regarding nutrition and you institution.

Results:

Line 148-150: with this statement I am assuming you used total motor scores, but it’s until now that I realize this. Please specify form the beginning and adjust accordingly.

If you used SCIM III version, please specify from the very beginning.

Line 153-155: Please check that the ranges in parentheses are correct: “The mean change in 154 SCIM score was 21.6 ± 18.22 (range, −2 – 80), with a mean SCIM recovery rate of 27.96 ± 155 24.84% (range, −2.38% – 95.74%) (Table 1)”

Line 161-168: I suggest adding all the percentages.

The paragraph on “Nutritional status at 4 weeks and 6 months after SCI”….is a bit confusing, try to present the results in order for pre and post by each outcome measure, other wise the reader has to move up and down to see the pre and post values.

The “Multivariable linear regression analyses for neurological and functional recovery” paragraph is a bit hard to follow; please consider re-ordering the information and avoiding repetition.

Conclusions:

This sentence in conclusions: “Although nutritional status was not an independent predictor of functional recovery assessed by SCI after multivariable adjustment, its significant associations with neurological improvement suggest that nutritional status may influence functional recovery indirectly through its effects on neurological recovery.” Belongs to the discussion, I don’t think this is a conclusion

Tables:

Could table 4 and 5 be condensed in one table?

Reviewer #2: Congratulations.

A very interesting cohort study correlating nutritional status with cervical spinal cord injury.

I have a question: among the patients included in this clinical research, were there any subject with a metabolic or endocrine disorder?

It would also be interesting to continue this type of study and include nutritional management during the follow-up period as a controlled variable.

Reviewer #3: In this retrospective study, the authors investigated the prognostic value of early nutritional indices - the Prognostic Nutritional Index (PNI), Geriatric Nutritional Risk Index (GNRI), and Controlling Nutritional Status (CONUT) score - in predicting recovery outcomes for patients with traumatic cervical spinal cord injury (SCI). The study highlights a modifiable factor in rehabilitation of patients with cervical spinal cord injury. The authors have utilized appropriate multivariate liner regression tools to control for confounders. The manuscript is well-structured, and the results have been compiled appropriately. However, the following major and minor flaws remain that need to be addressed:

1. Methods: Please explain how 4 weeks post injury time interval was selected as “early nutritional status” in this study vs. on admission? Is this a standard timeline for determining early nutritional status in spinal cord injury patients? Discuss the rationale for selecting this cut off in the methods.

2. What was the mean length of stay for 206 eligible patients?

3. The study started with 206 eligible patients but excluded 94 due to early discharge/transfer. The authors must address whether the "early discharge" group differed significantly from the "included" group. If the healthier patients were discharged early, the remaining cohort might be skewed toward more severe cases, biasing the results.

4. Serum albumin is a negative acute phase reactant in critically ill patients and hence may influence PNI. A low PNI may simply be a proxy for a high "complication burden" (pneumonia, UTI) rather than nutritional intake itself. Please discuss under limitations of using PNI as a nutritional index.

5. The study did not mention the type of nutritional support received (TPN, enteral, or oral) during the first few weeks post injury. Differences in feeds and mode of providing nutrition could be a significant confounding variable that influences both the PNI and the recovery rate. Please address this under Discussion.

6. In the Results (Line 162), the PNI categories are listed as "good, normal, and poor," while the Methods (Line 90) use "normal, mild, and moderate to severe." Please check and make necessary corrections to ensure consistency throughout the manuscript including tables.

7. Due to retrospective nature of the study, poor nutritional status as a cause for poor neurological recovery cannot be determined. Low PNI may represent an inflammatory state especially in patients with complications (pneumonia, pressure ulcers, etc.) post injury. The conclusions should explicitly suggest that PNI may be a useful prognostic marker for neurological and functional recovery.

**********

6. PLOS authors have the option to publish the peer review history of their article (what does this mean?). If published, this will include your full peer review and any attached files.

If you choose “no”, your identity will remain anonymous but your review may still be made public.

**Do you want your identity to be public for this peer review?**  For information about this choice, including consent withdrawal, please see our Privacy Policy.

Reviewer #1: No

Reviewer #2: No

Reviewer #3: No

**********

[NOTE: If reviewer comments were submitted as an attachment file, they will be attached to this email and accessible via the submission site. Please log into your account, locate the manuscript record, and check for the action link "View Attachments". If this link does not appear, there are no attachment files.]

To ensure your figures meet our technical requirements, please review our figure guidelines: https://journals.plos.org/plosone/s/figures

You may also use PLOS’s free figure tool, NAAS, to help you prepare publication quality figures: https://journals.plos.org/plosone/s/figures#loc-tools-for-figure-preparation.

NAAS will assess whether your figures meet our technical requirements by comparing each figure against our figure specifications.

# Author comment: Author response to Decision Letter 1

**DOI:** 10.1371/journal.pone.0353302.r002

19 May 2026

To the editor and reviewers:

We appreciate the high quality and constructive review given to our manuscript. We have made every effort to carefully address all comments and suggestions in a point-by-point manner, as we believe they have helped to improve the clarity and scientific quality of our work. We would be pleased to provide further clarification should any points require additional explanation. Detailed responses to each comment are provided in the attached “Response to Reviewers” file.

Attachment Submitted filename: Response to Reviewers.docx

# Peer review: Decision Letter 1

**DOI:** 10.1371/journal.pone.0353302.r003

**Authors:** Zubing Mei

22 Jun 2026

Prognostic Significance of Nutritional Status for Neurological and Functional Recovery after Cervical Spinal Cord Injury

PONE-D-26-09186R1

Dear Dr. Yokota,

We’re pleased to inform you that your manuscript has been judged scientifically suitable for publication and will be formally accepted for publication once it meets all outstanding technical requirements.

Within one week, you’ll receive an e-mail detailing the required amendments. When these have been addressed, you’ll receive a formal acceptance letter and your manuscript will be scheduled for publication.

An invoice will be generated when your article is formally accepted. Please note, if your institution has a publishing partnership with PLOS and your article meets the relevant criteria, all or part of your publication costs will be covered. Please make sure your user information is up-to-date by logging into Editorial Manager at Editorial Manager® and clicking the ‘Update My Information' link at the top of the page. For questions related to billing, please contact billing support.

If your institution or institutions have a press office, please notify them about your upcoming paper to help maximize its impact. If they’ll be preparing press materials, please inform our press team as soon as possible -- no later than 48 hours after receiving the formal acceptance. Your manuscript will remain under strict press embargo until 2 pm Eastern Time on the date of publication. For more information, please contact onepress@plos.org.

Kind regards,

Zubing Mei, MD,Ph.D

Academic Editor

PLOS One

Additional Editor Comments (optional):

Reviewers' comments:

Reviewer's Responses to Questions

**Comments to the Author**

1. If the authors have adequately addressed your comments raised in a previous round of review and you feel that this manuscript is now acceptable for publication, you may indicate that here to bypass the “Comments to the Author” section, enter your conflict of interest statement in the “Confidential to Editor” section, and submit your "Accept" recommendation.

Reviewer #3: All comments have been addressed

**********

2. Is the manuscript technically sound, and do the data support the conclusions?

The manuscript must describe a technically sound piece of scientific research with data that supports the conclusions. Experiments must have been conducted rigorously, with appropriate controls, replication, and sample sizes. The conclusions must be drawn appropriately based on the data presented.

Reviewer #3: Yes

**********

3. Has the statistical analysis been performed appropriately and rigorously?

Reviewer #3: Yes

**********

4. Have the authors made all data underlying the findings in their manuscript fully available?

The PLOS Data policy requires authors to make all data underlying the findings described in their manuscript fully available without restriction, with rare exception (please refer to the Data Availability Statement in the manuscript PDF file). The data should be provided as part of the manuscript or its supporting information, or deposited to a public repository. For example, in addition to summary statistics, the data points behind means, medians and variance measures should be available. If there are restrictions on publicly sharing data—e.g. participant privacy or use of data from a third party—those must be specified.

Reviewer #3: Yes

**********

5. Is the manuscript presented in an intelligible fashion and written in standard English?

PLOS ONE does not copyedit accepted manuscripts, so the language in submitted articles must be clear, correct, and unambiguous. Any typographical or grammatical errors should be corrected at revision, so please note any specific errors here.

Reviewer #3: Yes

**********

6. Review Comments to the Author

Please use the space provided to explain your answers to the questions above. You may also include additional comments for the author, including concerns about dual publication, research ethics, or publication ethics. (Please upload your review as an attachment if it exceeds 20,000 characters)

Reviewer #3: (No Response)

**********

7. PLOS authors have the option to publish the peer review history of their article (what does this mean?). If published, this will include your full peer review and any attached files.

If you choose “no”, your identity will remain anonymous but your review may still be made public.

**Do you want your identity to be public for this peer review?**  For information about this choice, including consent withdrawal, please see our Privacy Policy.

Reviewer #3: No

**********

# Editor report: Acceptance letter

**DOI:** 10.1371/journal.pone.0353302.r004

**Authors:** Zubing Mei

PONE-D-26-09186R1

PLOS One

Dear Dr. Yokota,

I'm pleased to inform you that your manuscript has been deemed suitable for publication in PLOS One. Congratulations! Your manuscript is now being handed over to our production team.

At this stage, our production department will prepare your paper for publication. This includes ensuring the following:

* All references, tables, and figures are properly cited

* All relevant supporting information is included in the manuscript submission,

* There are no issues that prevent the paper from being properly typeset

You will receive further instructions from the production team, including instructions on how to review your proof when it is ready. Please keep in mind that we are working through a large volume of accepted articles, so please give us a few days to review your paper and let you know the next and final steps.

Lastly, if your institution or institutions have a press office, please let them know about your upcoming paper now to help maximize its impact. If they'll be preparing press materials, please inform our press team within the next 48 hours. Your manuscript will remain under strict press embargo until 2 pm Eastern Time on the date of publication. For more information, please contact onepress@plos.org.

You will receive an invoice from PLOS for your publication fee after your manuscript has reached the completed accept phase. If you receive an email requesting payment before acceptance or for any other service, this may be a phishing scheme. Learn how to identify phishing emails and protect your accounts at https://explore.plos.org/phishing.

If we can help with anything else, please email us at customercare@plos.org.

Thank you for submitting your work to PLOS ONE and supporting open access.

Kind regards,

PLOS ONE Editorial Office Staff

on behalf of

Dr. Zubing Mei

Academic Editor

PLOS One
